# Supplementary material for: Genomic epidemiology of Plasmodium knowlesi reveals putative genetic drivers of adaptation in Malaysia
Source: PLoS Negl Trop Dis. 2025 Mar 12;19(3):e0012885. doi: 10.1371/journal.pntd.0012885 (PMC11932472; doi:10.1371/journal.pntd.0012885)
Supplement: S1 Text — (DOCX) [file pntd.0012885.s001.docx]

**Genomic epidemiology of *Plasmodium knowlesi* reveals putative genetic drivers of adaptation in Malaysia.**

Jacob A F Westaway^*, 1, 2^, Ernest Diez Benavente^3^, Sarah Auburn^1^, Michal Kucharski^4^, Nicolas Aranciaga^4^, Sourav Nayak^4^, Timothy William^5^, Giri S Rajahram^5,6^, Kim A Piera^1^, Kamil Braima^1^, Angelica F Tan^1^, Danshy A. Alaza^5^, Bridget E Barber^1^, Chris Drakeley^7^, Roberto Amato^8^, Edwin Sutanto^11^, Hidayat Trimarsanto^1,12^, Jenarun Jelip^13^, Nicholas M Anstey^1^, Zbynek Bozdech^4^, Matthew Field^†, 1,2,9,10^, Matthew J Grigg^†, 1^

* Jacob.westaway@menzies.edu.au

^†^Equal contribution

## Affiliations

^1^ Global and Tropical Health, Menzies School of Health Research and Charles Darwin University, Darwin, Northern Territory, Australia

^2^ Centre for Tropical Bioinformatics and Molecular Biology, James Cook University, Cairns, Queensland, Australia

^3^ Laboratory of Experimental Cardiology, University Medical Center Utrecht, Utrecht, the Netherlands

^4^ Nanyang Technological University, Singapore

^5^ Infectious Disease Society Kota Kinabalu, Kota Kinabalu, Sabah, Malaysia

^6^ Queen Elizabeth II Hospital-Clinical Research Centre, Ministry of Health, Kota Kinabalu, Sabah, Malaysia

^7^ London School of Hygiene & Tropical Medicine, London, United Kingdom

^8^ Wellcome Sanger Institute, Hinxton, United Kingdom

^9^ College of Public Health, Medical and Veterinary Science, James Cook University, Townsville, Queensland, Australia

^10^ Immunogenomics Lab, Garvan Institute of Medical Research, Darlinghurst, New South Wales, Australia

^11^ Exeins Health Initiative, Jakarta, Indonesia

^12^ Eijkman Research Center for Molecular Biology, National Research and Innovation Agency, Jakarta, Indonesia

^13^ Universiti Malaysia Sabah, Kota Kinabalu, Sabah, Malaysia


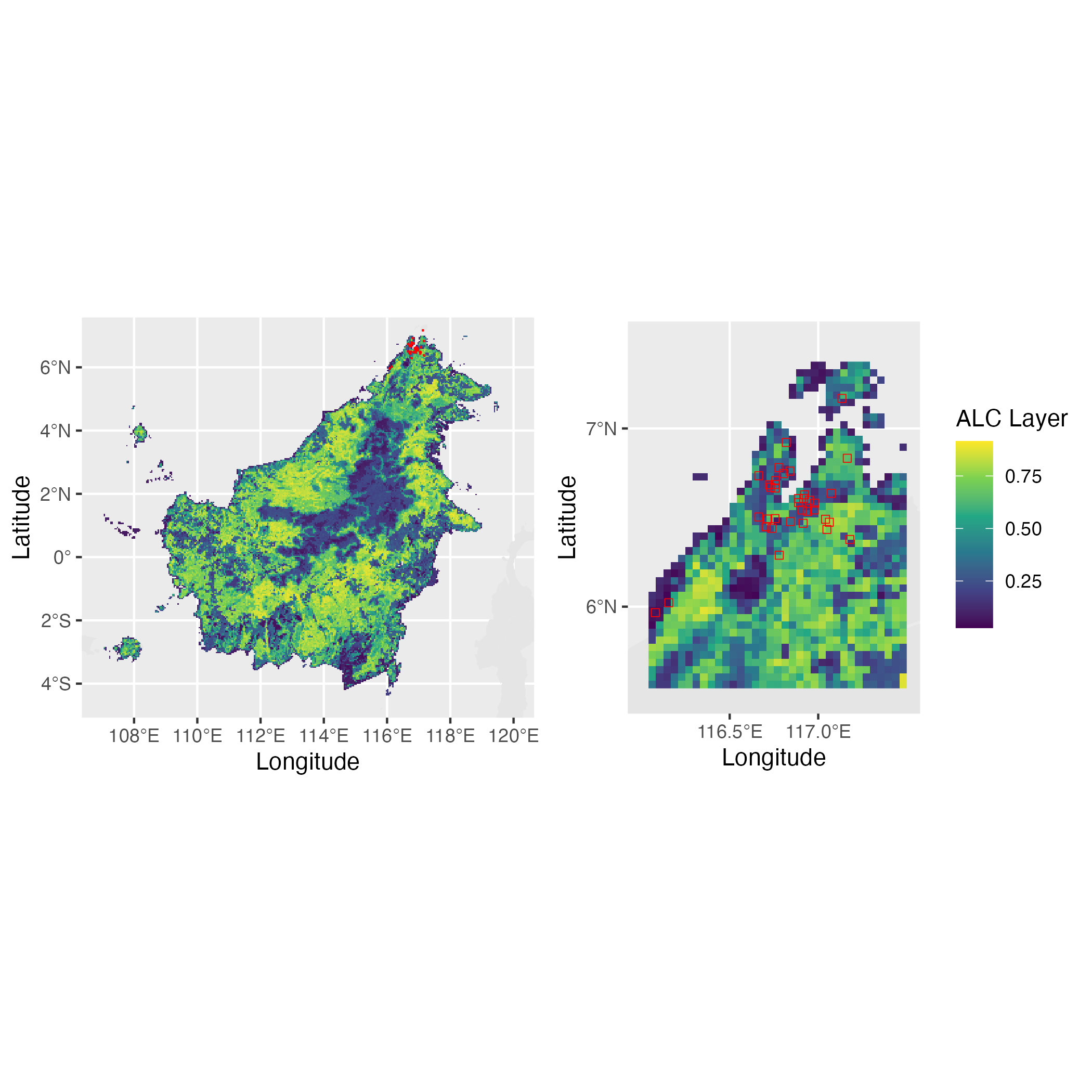


**Supplementary Figure A.** Maps with raster densities representing the predicted distributions of the *Anopheles* Leucosphyrus Complex and overlayed with 5km x 5km grids around the Geographic Information System (GIS) data points for samples in the ecological analysis. Abbreviations; ALC: *Anopheles* Leucosphyrus Complex. Shapefile made with Natural Earth: https://www.naturalearthdata.com/downloads/50m-cultural-vectors/50m-admin-1-states-provinces/.


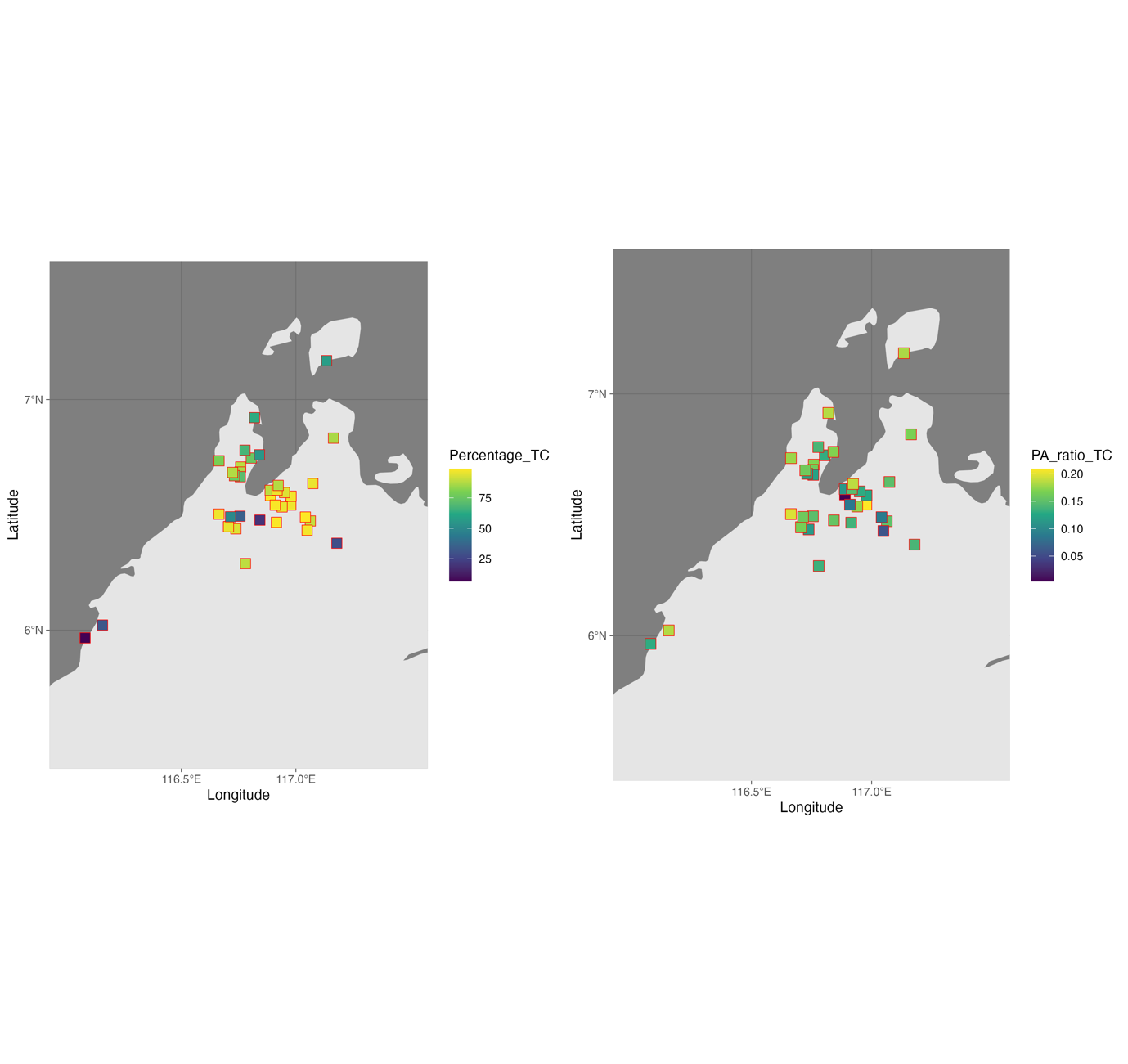


**Supplementary Figure B.** Maps with densities representing the landscape data (percentage of landscape on the left and perimeter-area ratio on the right) and overlayed with 5km x 5km grids around the GIS data points for samples in the ecological analysis. Abbreviations; Percentage_TC: percentage of landscape, PA_ratio_TC: perimeter-area ratio. Shapefile made with Natural Earth: https://www.naturalearthdata.com/downloads/50m-cultural-vectors/50m-admin-1-states-provinces/.


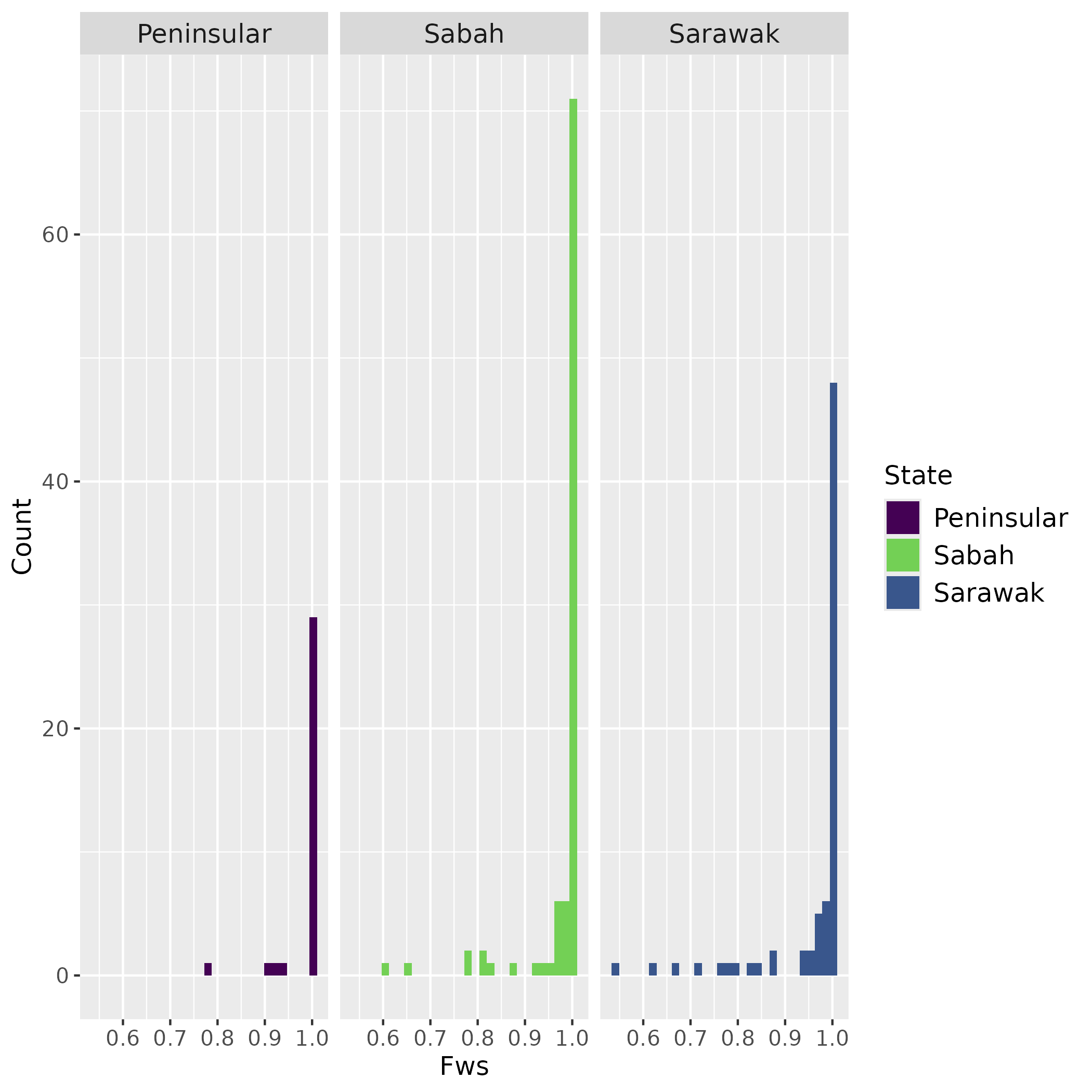


**Supplementary Figure C.** Histogram of showing the significant difference between F_ws_ values in each of the states (*ꭓ^2^* = 41.58, df = 2, p < 0.01). Sarawak had significantly lower F_ws_ than both Sabah (p < 0.05) and Peninsular Malaysia (p < 0.05).


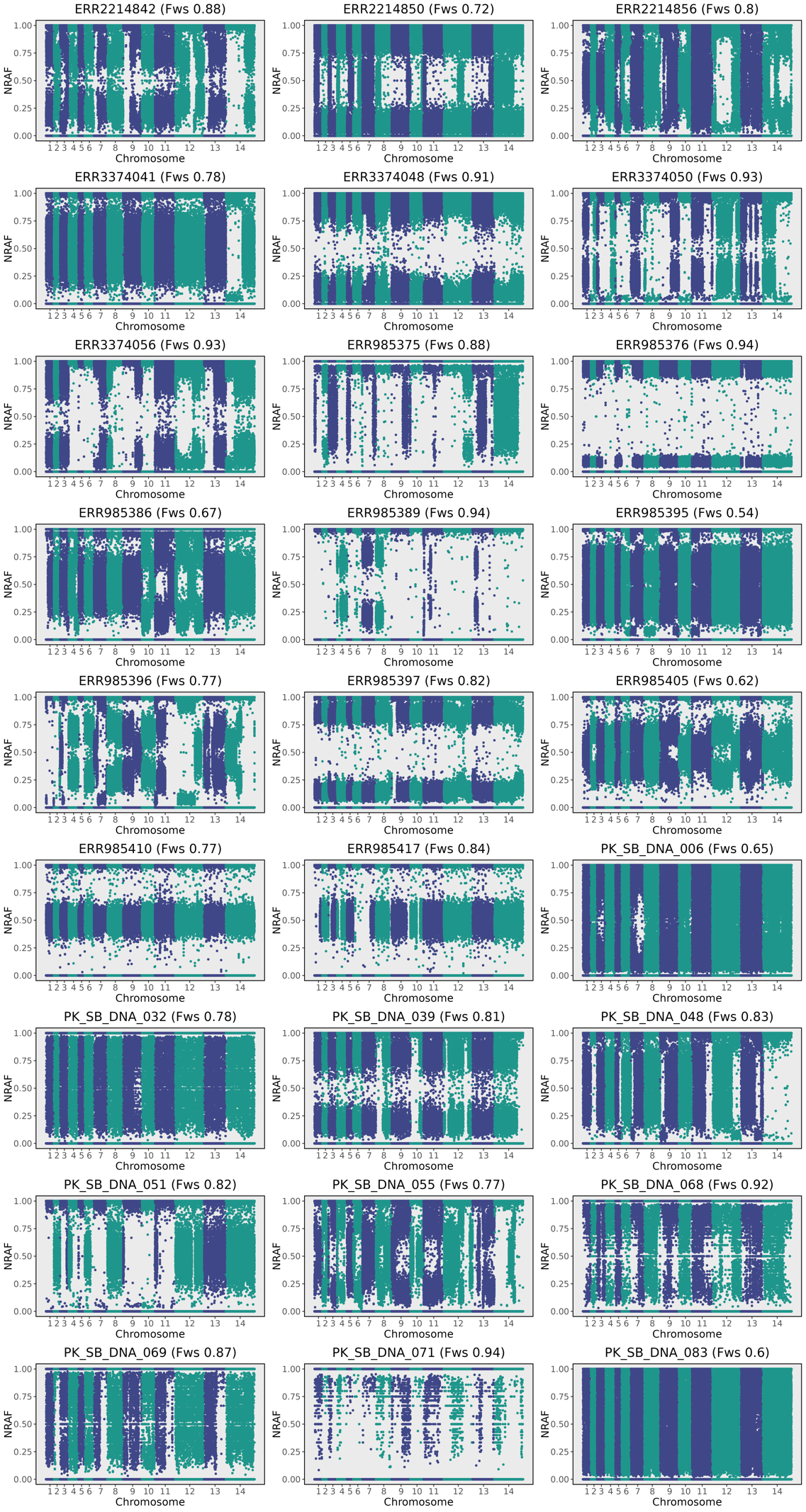


**Supplementary Figure D.** Manhattan plots of the within-isolate non-reference allele frequencies (NRAF) across the genome for polyclonal (*F_WS_* < 0.95.) samples.


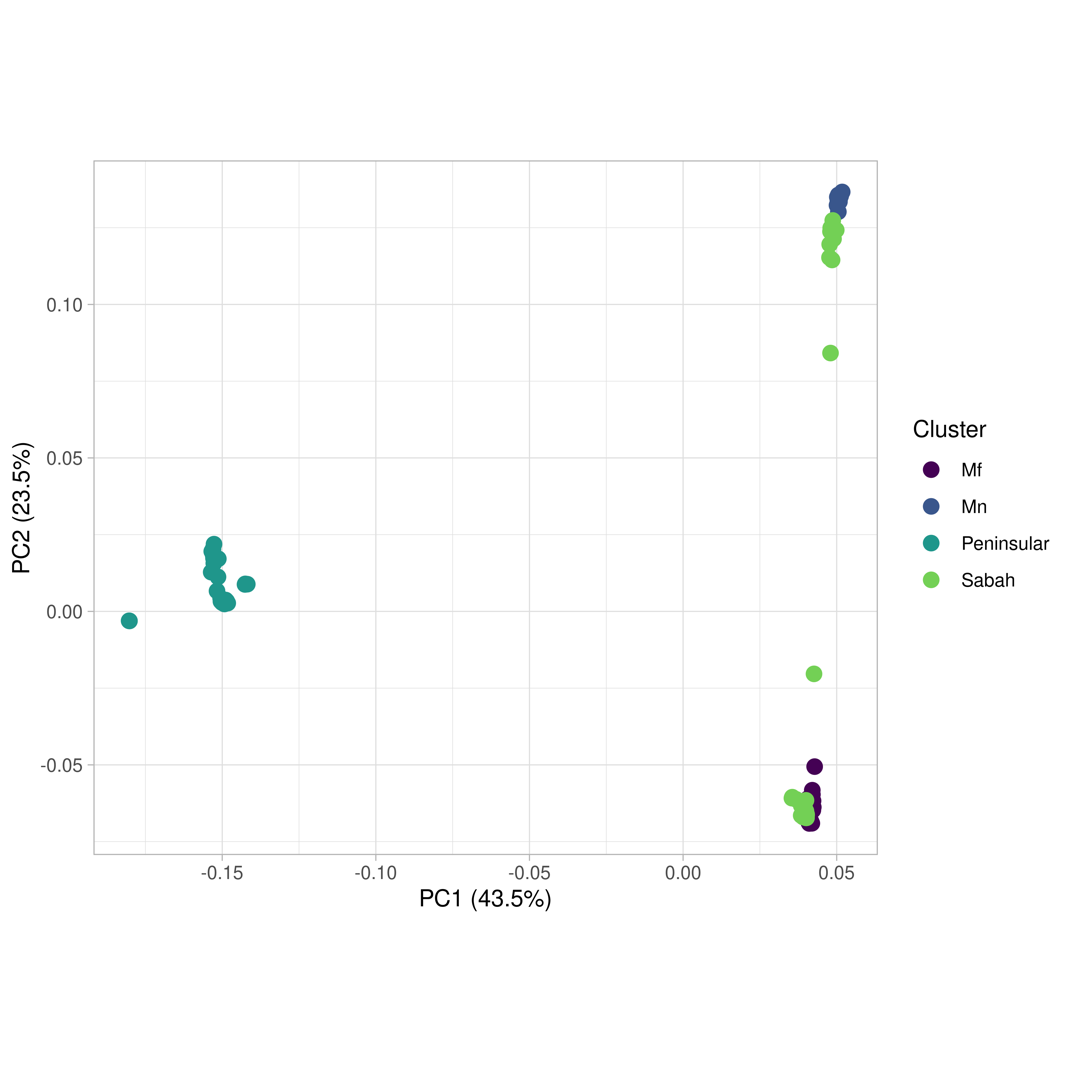


**Supplementary Figure E.** Principle component analysis depicting three predominant genomic clusters of *P. knowlesi* across Malaysia, specifically the Peninsular Malaysia sub-population (*Peninsular*), and Malaysian-Borneo macaque-associated subpopulations of Macaca fascicularis (Mf) and M. nemestrina (*Mn*). The new isolates from Sabah are labelled separately (*Sabah*).


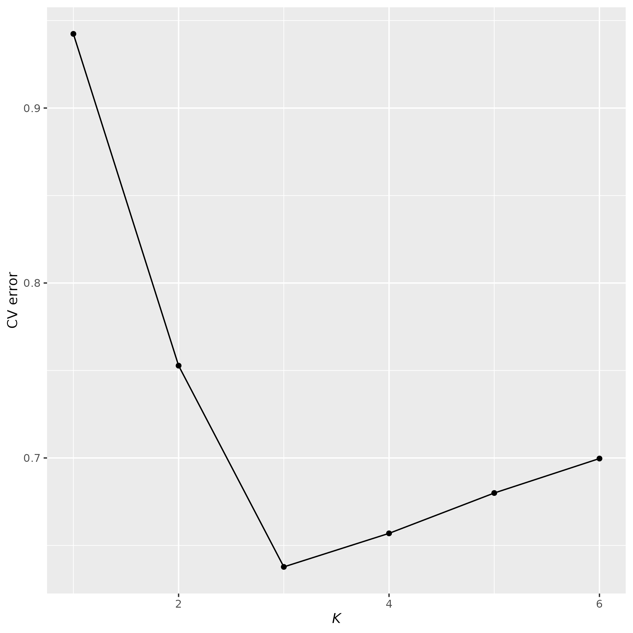


**Supplementary Figure F.** Admixture cross-validation errors for estimates of K 1 to 6 in the country-wide sample set.

**
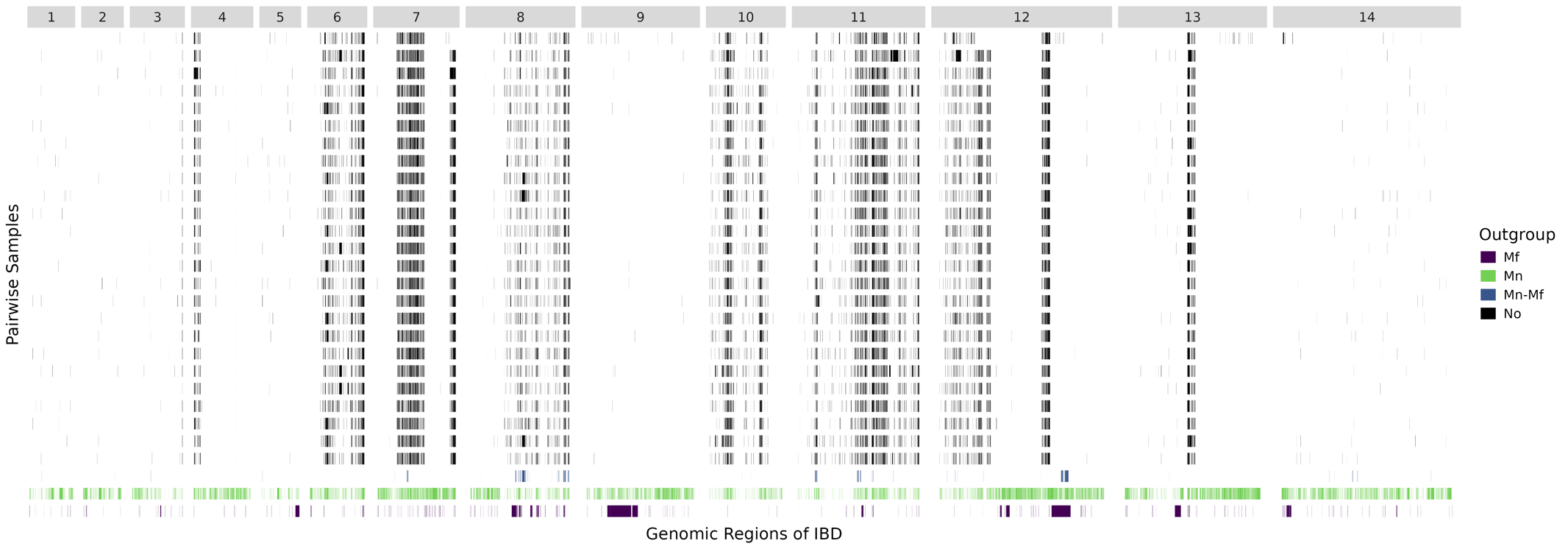
**

**Supplementary Figure G.** Segmented column plot showing similar regions of identity-by-decent (IBD) across the genome for pairwise comparisons of a single *Mf* isolate and a collection of *Mn* isolates that share >10% IBD (Outgroup = No). At a 10% IBD threshold, this *Mf* isolate has no connectivity to other *Mf* isolates. Three outgroups are also presented: pairwise *Mn* isolates that share 47% IBD (Outgroup = Mn), pairwise *Mf* isolates that share ~11% IBD (Outgroup = Mf), and an Mf-to-Mn pairwise comparison that shares ~2% IBD (Outgroup = Mn-Mf).


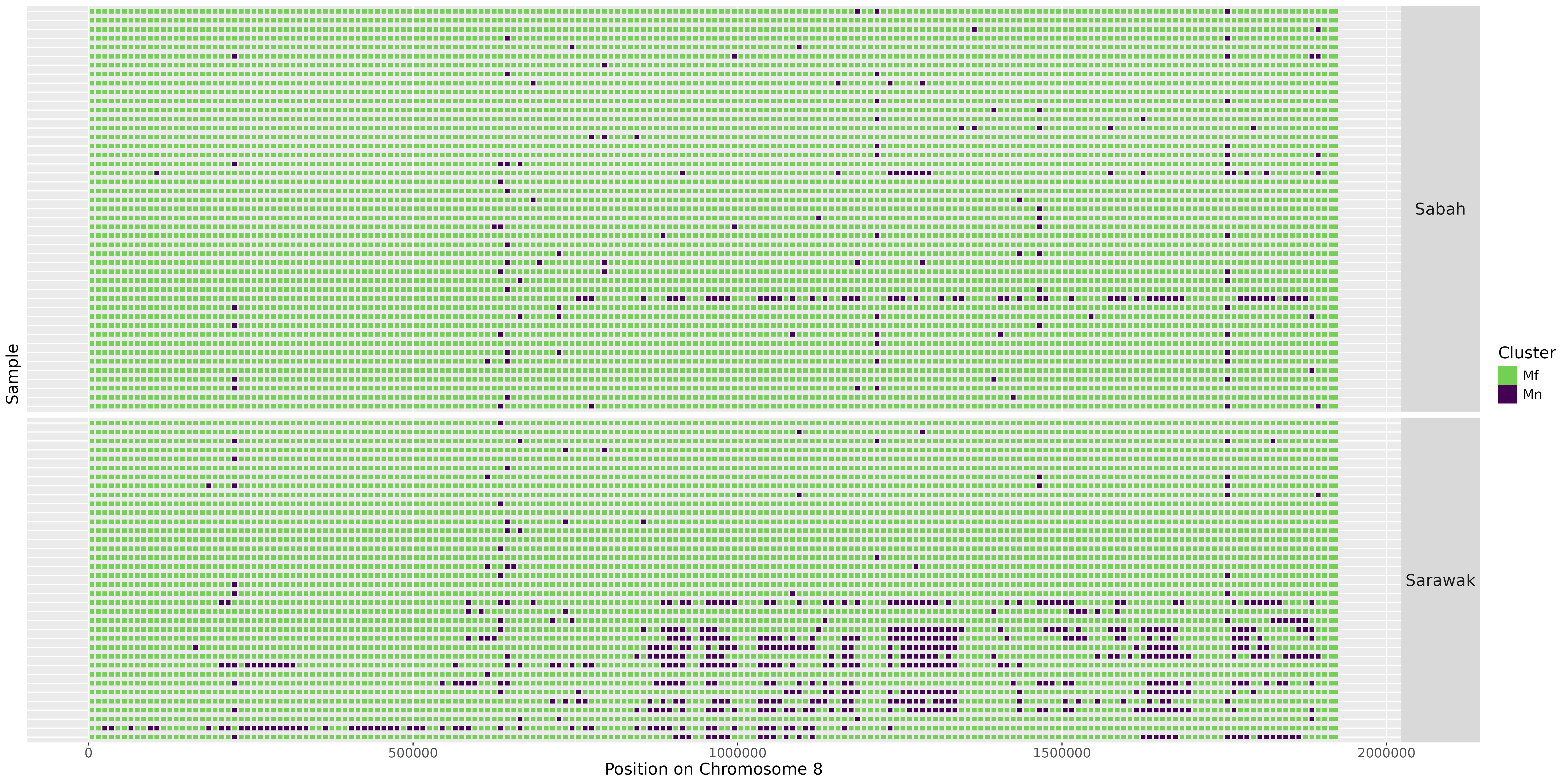


**Supplementary Figure H.** Tile plot of introgression windows (10kb) across chromosome 8 for all *Mf* samples, where introgressed windows are designated *Mn* (more genetically similar to *Mn* than *Mf*), demonstrating the level of fragmentation across highly introgressed regions and highlighting the difficulty in assigning biological windows.


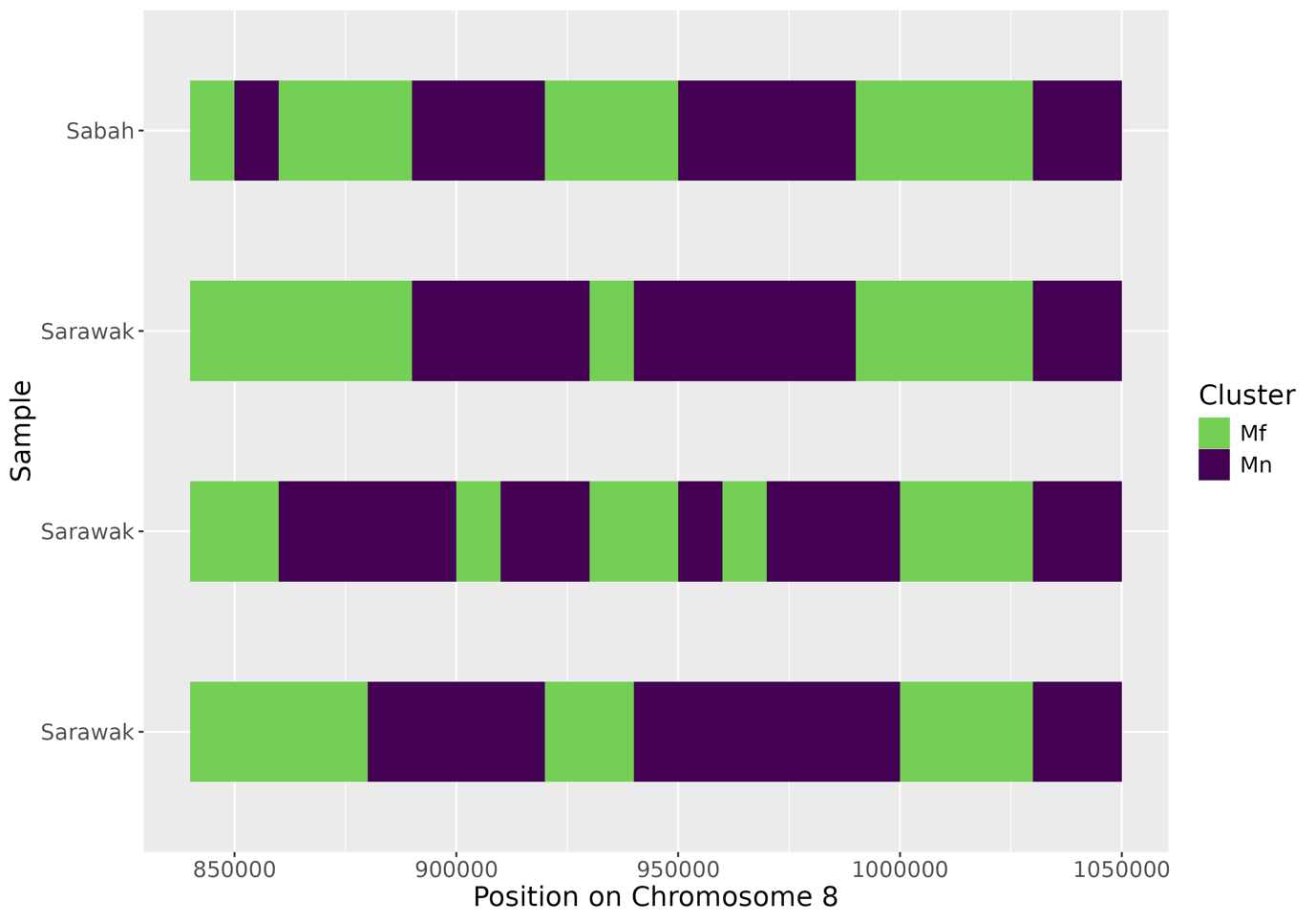


**Supplementary Figure I.** Column plot of introgressed windows (10kb) on a sub-section of chromosome 8 across one Sabah and several Sarawak isolates, showing that the introgressed windows do not perfectly overlap, and thus, the breakpoints are likely to be different. This supports the hypothesis that similar introgressions could be arising independently in different locations (Sabah and Sarawak).

| Sample | State | District | Cluster | Proportion |
| --- | --- | --- | --- | --- |
| ERR2214837 | Sarawak | Kapit | Mn | 0.998 |
| ERR2214838 | Sarawak | Kapit | Mn | 0.997 |
| ERR2214839 | Sarawak | Kapit | Mn | 0.998 |
| ERR2214840 | Sarawak | Kapit | Mn | 0.998 |
| ERR2214841 | Sarawak | Kapit | Mn | 0.997 |
| ERR2214842 | Sarawak | Kapit | Mn | 0.877 |
| ERR2214843 | Sarawak | Kapit | Mn | 0.998 |
| ERR2214844 | Sarawak | Kapit | Mn | 0.998 |
| ERR2214845 | Sarawak | Kapit | Mn | 0.969 |
| ERR2214846 | Sarawak | Kapit | Mn | 0.998 |
| ERR2214847 | Sarawak | Kapit | Mn | 0.997 |
| ERR2214848 | Sarawak | Kapit | Mn | 0.997 |
| ERR2214849 | Sarawak | Kapit | Mn | 0.997 |
| ERR2214850 | Sarawak | Kapit | NA | 0.724 |
| ERR2214851 | Sarawak | Kapit | Mn | 0.997 |
| ERR2214852 | Sarawak | Kapit | Mn | 0.997 |
| ERR2214853 | Sarawak | Kapit | Mn | 0.998 |
| ERR2214854 | Sarawak | Kapit | Mn | 0.997 |
| ERR2214855 | Sarawak | Kapit | Mn | 0.997 |
| ERR2214856 | Sarawak | Kapit | NA | 0.8 |
| ERR2214857 | Sarawak | Kapit | Mn | 0.989 |
| ERR274221 | Sarawak | Sarikei | Mf | 0.978 |
| ERR274222 | Sarawak | Sarikei | Mf | 0.997 |
| ERR274224 | Sarawak | Sarikei | Mn | 0.997 |
| ERR274225 | Sarawak | Sarikei | Mn | 0.997 |
| ERR3374031 | Kelantan | Gua Musang | Peninsular | 0.998 |
| ERR3374032 | Kelantan | Gua Musang | Peninsular | 0.998 |
| ERR3374033 | Kelantan | Gua Musang | Peninsular | 0.997 |
| ERR3374034 | Kelantan | Gua Musang | Peninsular | 0.998 |
| ERR3374035 | Pahang | Kuala Lipis | Peninsular | 0.998 |
| ERR3374036 | Pahang | Kuala Lipis | Peninsular | 0.999 |
| ERR3374037 | Pahang | Kuala Lipis | Peninsular | 0.998 |
| ERR3374038 | Pahang | Kuala Lipis | Peninsular | 0.998 |
| ERR3374039 | Pahang | Kuala Lipis | Peninsular | 0.998 |
| ERR3374040 | Pahang | Kuala Lipis | Peninsular | 0.999 |
| ERR3374041 | Pahang | Kuala Lipis | NA | 0.782 |
| ERR3374042 | Pahang | Kuala Lipis | Peninsular | 0.998 |
| ERR3374043 | Pahang | Kuala Lipis | Peninsular | 0.998 |
| ERR3374044 | Pahang | Kuala Lipis | Peninsular | 0.998 |
| ERR3374045 | Pahang | Kuala Lipis | Peninsular | 0.998 |
| ERR3374046 | Pahang | Kuala Lipis | Peninsular | 0.998 |
| ERR3374047 | Pahang | Kuala Lipis | Peninsular | 0.999 |
| ERR3374048 | Pahang | Kuala Lipis | Peninsular | 0.914 |
| ERR3374049 | Pahang | Kuala Lipis | Peninsular | 0.999 |
| ERR3374050 | Pahang | Kuala Lipis | Peninsular | 0.928 |
| ERR3374051 | Perak | Sungai Siput | Peninsular | 0.998 |
| ERR3374052 | Perak | Sungai Siput | Peninsular | 0.998 |
| ERR3374053 | Perak | Sungai Siput | Peninsular | 0.996 |
| ERR3374054 | Perak | Sungai Siput | Peninsular | 0.999 |
| ERR3374055 | Pahang | Temerloh | Peninsular | 0.998 |
| ERR3374056 | Pahang | Temerloh | Peninsular | 0.932 |
| ERR3374057 | Perak | Taiping | Peninsular | 0.998 |
| ERR3374058 | Perak | Taiping | Peninsular | 0.998 |
| ERR366425 | Sarawak | Sarikei | Mn | 0.993 |
| ERR366426 | Sarawak | Sarikei | Mf | 0.998 |
| ERR985372 | Sarawak | Betong | Mf | 0.998 |
| ERR985373 | Sarawak | Betong | Mf | 0.997 |
| ERR985374 | Sarawak | Betong | Mf | 0.997 |
| ERR985375 | Sarawak | Betong | Mf | 0.877 |
| ERR985376 | Sarawak | Betong | Mf | 0.937 |
| ERR985377 | Sarawak | Betong | Mf | 0.998 |
| ERR985378 | Sarawak | Betong | Mf | 0.998 |
| ERR985379 | Sarawak | Betong | Mf | 0.997 |
| ERR985380 | Sarawak | Betong | Mf | 0.972 |
| ERR985381 | Sarawak | Betong | Mf | 0.998 |
| ERR985382 | Sarawak | Betong | Mf | 0.991 |
| ERR985383 | Sarawak | Betong | Mf | 0.985 |
| ERR985384 | Sarawak | Betong | Mf | 0.988 |
| ERR985385 | Sarawak | Kapit | Mf | 0.997 |
| ERR985386 | Sarawak | Kapit | NA | 0.671 |
| ERR985387 | Sarawak | Kapit | Mf | 0.972 |
| ERR985388 | Sarawak | Kapit | Mf | 0.997 |
| ERR985389 | Sarawak | Kapit | Mf | 0.941 |
| ERR985390 | Sarawak | Kapit | Mf | 0.996 |
| ERR985391 | Sarawak | Kapit | Mf | 0.998 |
| ERR985392 | Sarawak | Kapit | Mf | 0.998 |
| ERR985393 | Sarawak | Kapit | Mf | 0.984 |
| ERR985394 | Sarawak | Kapit | Mf | 0.998 |
| ERR985395 | Sarawak | Kapit | NA | 0.539 |
| ERR985396 | Sarawak | Kapit | NA | 0.774 |
| ERR985397 | Sarawak | Kapit | NA | 0.824 |
| ERR985398 | Sarawak | Kapit | Mf | 0.998 |
| ERR985399 | Sarawak | Kapit | Mf | 0.998 |
| ERR985400 | Sarawak | Kapit | Mf | 0.997 |
| ERR985401 | Sarawak | Kapit | Mf | 0.998 |
| ERR985402 | Sarawak | Kapit | Mf | 0.997 |
| ERR985403 | Sarawak | Kapit | Mf | 0.998 |
| ERR985404 | Sarawak | Kapit | Mf | 0.969 |
| ERR985405 | Sarawak | Kapit | NA | 0.625 |
| ERR985406 | Sarawak | Kapit | Mf | 0.998 |
| ERR985407 | Sarawak | Kapit | Mf | 0.998 |
| ERR985408 | Sarawak | Kapit | Mf | 0.998 |
| ERR985409 | Sarawak | Kapit | Mf | 0.956 |
| ERR985410 | Sarawak | Betong | NA | 0.771 |
| ERR985411 | Sarawak | Betong | Mn | 0.996 |
| ERR985412 | Sarawak | Kapit | Mn | 0.997 |
| ERR985413 | Sarawak | Kapit | Mn | 0.998 |
| ERR985415 | Sarawak | Kapit | Mn | 0.997 |
| ERR985416 | Sarawak | Kapit | Mn | 0.998 |
| ERR985417 | Sarawak | Kapit | NA | 0.84 |
| ERR985418 | Sarawak | Kapit | Mn | 0.998 |
| ERR985419 | Sarawak | Kapit | Mn | 0.956 |
| PK_SB_DNA_001 | Sabah | Kota Belud | Mf | 0.999 |
| PK_SB_DNA_002 | Sabah | Kota Kinabalu | Mf | 0.998 |
| PK_SB_DNA_003 | Sabah | Kudat | Mf | 0.998 |
| PK_SB_DNA_004 | Sabah | Sabah | Mf | 1 |
| PK_SB_DNA_005 | Sabah | Kudat | Mf | 0.998 |
| PK_SB_DNA_006 | Sabah | Pitas | NA | 0.652 |
| PK_SB_DNA_007 | Sabah | Sabah | Mf | 0.979 |
| PK_SB_DNA_008 | Sabah | Kota Kinabalu | Mf | 0.999 |
| PK_SB_DNA_009 | Sabah | Tuaran | Mf | 0.998 |
| PK_SB_DNA_010 | Sabah | Kudat | Mf | 0.954 |
| PK_SB_DNA_011 | Sabah | Papar | Mn | 0.972 |
| PK_SB_DNA_012 | Sabah | Sabah | Mf | 0.986 |
| PK_SB_DNA_013 | Sabah | Kudat | Mf | 0.998 |
| PK_SB_DNA_014 | Sabah | Ranau | Mf | 0.998 |
| PK_SB_DNA_015 | Sabah | Kudat | Mf | 0.998 |
| PK_SB_DNA_016 | Sabah | Papar | Mn | 0.997 |
| PK_SB_DNA_017 | Sabah | Sabah | Mf | 0.999 |
| PK_SB_DNA_018 | Sabah | Sabah | Mf | 0.999 |
| PK_SB_DNA_019 | Sabah | Sabah | Mf | 1 |
| PK_SB_DNA_020 | Sabah | Sabah | Mf | 1 |
| PK_SB_DNA_021 | Sabah | Penampang | Mf | 0.998 |
| PK_SB_DNA_022 | Sabah | Sabah | Mf | 0.999 |
| PK_SB_DNA_023 | Sabah | Kudat | Mf | 0.998 |
| PK_SB_DNA_024 | Sabah | Sabah | Mf | 0.999 |
| PK_SB_DNA_025 | Sabah | Sabah | Mf | 0.973 |
| PK_SB_DNA_026 | Sabah | Sabah | Mf | 0.996 |
| PK_SB_DNA_027 | Sabah | Sabah | Mf | 0.998 |
| PK_SB_DNA_028 | Sabah | Papar | Mf | 0.998 |
| PK_SB_DNA_029 | Sabah | Kota Marudu | Mf | 0.998 |
| PK_SB_DNA_030 | Sabah | Kudat | Mn | 0.998 |
| PK_SB_DNA_031 | Sabah | Kudat | Mf | 0.998 |
| PK_SB_DNA_032 | Sabah | Tuaran | NA | 0.782 |
| PK_SB_DNA_033 | Sabah | Sabah | Mf | 0.999 |
| PK_SB_DNA_034 | Sabah | Pitas | Mf | 0.999 |
| PK_SB_DNA_035 | Sabah | Kota Marudu | Mf | 0.998 |
| PK_SB_DNA_036 | Sabah | Kota Marudu | Mf | 0.998 |
| PK_SB_DNA_037 | Sabah | Sabah | Mf | 0.999 |
| PK_SB_DNA_038 | Sabah | Kota Marudu | Mf | 0.998 |
| PK_SB_DNA_039 | Sabah | Pitas | NA | 0.81 |
| PK_SB_DNA_040 | Sabah | Pitas | Mf | 0.998 |
| PK_SB_DNA_041 | Sabah | Sabah | Mf | 1 |
| PK_SB_DNA_042 | Sabah | Ranau | Mn | 0.999 |
| PK_SB_DNA_043 | Sabah | Kota Marudu | Mn | 0.998 |
| PK_SB_DNA_044 | Sabah | Kota Marudu | Mf | 0.998 |
| PK_SB_DNA_045 | Sabah | Kota Marudu | Mf | 0.998 |
| PK_SB_DNA_046 | Sabah | Kota Marudu | Mf | 0.998 |
| PK_SB_DNA_047 | Sabah | Kota Marudu | Mf | 0.975 |
| PK_SB_DNA_048 | Sabah | Tenom | NA | 0.826 |
| PK_SB_DNA_049 | Sabah | Pitas | Mf | 0.998 |
| PK_SB_DNA_050 | Sabah | Sabah | Mf | 0.999 |
| PK_SB_DNA_051 | Sabah | Pitas | NA | 0.819 |
| PK_SB_DNA_052 | Sabah | Pitas | Mf | 0.998 |
| PK_SB_DNA_053 | Sabah | Kudat | Mf | 0.998 |
| PK_SB_DNA_054 | Sabah | Kudat | Mf | 0.998 |
| PK_SB_DNA_055 | Sabah | Pitas | NA | 0.774 |
| PK_SB_DNA_056 | Sabah | Sabah | Mf | 0.975 |
| PK_SB_DNA_057 | Sabah | Kota Marudu | Mf | 0.998 |
| PK_SB_DNA_058 | Sabah | Sabah | Mf | 0.986 |
| PK_SB_DNA_059 | Sabah | Sabah | Mf | 0.996 |
| PK_SB_DNA_060 | Sabah | Sabah | Mf | 1 |
| PK_SB_DNA_061 | Sabah | Sabah | Mf | 1 |
| PK_SB_DNA_062 | Sabah | Sabah | Mf | 0.969 |
| PK_SB_DNA_063 | Sabah | Ranau | Mn | 0.99 |
| PK_SB_DNA_064 | Sabah | Sabah | Mf | 1 |
| PK_SB_DNA_065 | Sabah | Sabah | Mf | 1 |
| PK_SB_DNA_066 | Sabah | Sabah | Mf | 0.999 |
| PK_SB_DNA_067 | Sabah | Kota Marudu | Mf | 0.991 |
| PK_SB_DNA_068 | Sabah | Kota Marudu | Mf | 0.922 |
| PK_SB_DNA_069 | Sabah | Kota Marudu | Mf | 0.869 |
| PK_SB_DNA_070 | Sabah | Kota Marudu | Mf | 0.998 |
| PK_SB_DNA_071 | Sabah | Sabah | Mf | 0.944 |
| PK_SB_DNA_072 | Sabah | Kota Marudu | Mf | 0.998 |
| PK_SB_DNA_073 | Sabah | Kudat | Mf | 0.995 |
| PK_SB_DNA_074 | Sabah | Kota Marudu | Mf | 0.998 |
| PK_SB_DNA_075 | Sabah | Sabah | Mf | 0.998 |
| PK_SB_DNA_076 | Sabah | Sabah | Mf | 0.995 |
| PK_SB_DNA_077 | Sabah | Kudat | Mf | 0.986 |
| PK_SB_DNA_078 | Sabah | Sabah | Mf | 0.998 |
| PK_SB_DNA_079 | Sabah | Sabah | Mf | 0.999 |
| PK_SB_DNA_080 | Sabah | Sabah | Mf | 1 |
| PK_SB_DNA_081 | Sabah | Kudat | Mf | 0.999 |
| PK_SB_DNA_082 | Sabah | Kudat | Mf | 0.998 |
| PK_SB_DNA_083 | Sabah | Kota Belud | NA | 0.598 |
| PK_SB_DNA_084 | Sabah | Sabah | Mf | 0.999 |
| PK_SB_DNA_085 | Sabah | Sabah | Mf | 0.999 |
| PK_SB_DNA_086 | Sabah | Kota Marudu | Mf | 0.998 |
| PK_SB_DNA_087 | Sabah | Pitas | Mf | 0.999 |
| PK_SB_DNA_088 | Sabah | Kota Marudu | Mf | 0.998 |
| PK_SB_DNA_089 | Sabah | Beluran | Mf | 0.975 |
| PK_SB_DNA_090 | Sabah | Kota Marudu | Mf | 0.998 |
| PK_SB_DNA_091 | Sabah | Pitas | Mn | 0.998 |
| PK_SB_DNA_092 | Sabah | Kota Marudu | Mn | 0.998 |
| PK_SB_DNA_093 | Sabah | Kota Marudu | Mn | 0.999 |
| PK_SB_DNA_094 | Sabah | Kota Marudu | Mf | 0.998 |
| SRR2221468 | Peninsular | Clinic | Peninsular | 0.997 |
| SRR2222335 | Peninsular | Clinic | Peninsular | 1 |
| SRR2225467 | Peninsular | Clinic | Peninsular | 1 |
| SRR2225571 | Peninsular | Clinic | Peninsular | 0.998 |
| SRR2225573 | Peninsular | Clinic | Peninsular | 0.998 |
| SRR3135172 | Peninsular | Clinic | Peninsular | 1 |

**Supplementary Table A.** Sample names (accession numbers for publicly available data), geographic locations, designated *Plasmodium knowlesi* clusters (determined through neighbour-joining tree analyses) and genotype complexity (within-isolate fixation index [*F_WS_*]). Publicly available data are from the following publications: <https://doi.org/10.1038/s41598-019-46398-z> & <https://doi.org/10.3201/eid2608.190864>.

| ordered_PKA1H_01_v2 | 8541 | 20666 |
| --- | --- | --- |
| ordered_PKA1H_01_v2 | 25011 | 27638 |
| ordered_PKA1H_01_v2 | 163982 | 179200 |
| ordered_PKA1H_01_v2 | 182684 | 184429 |
| ordered_PKA1H_01_v2 | 610275 | 620884 |
| ordered_PKA1H_01_v2 | 622466 | 624727 |
| ordered_PKA1H_01_v2 | 628887 | 631002 |
| ordered_PKA1H_01_v2 | 864542 | 867967 |
| ordered_PKA1H_01_v2 | 872321 | 873587 |
| ordered_PKA1H_01_v2 | 888222 | 889978 |
| ordered_PKA1H_02_v2 | 523 | 3366 |
| ordered_PKA1H_02_v2 | 8570 | 14723 |
| ordered_PKA1H_02_v2 | 31368 | 35065 |
| ordered_PKA1H_02_v2 | 132700 | 135317 |
| ordered_PKA1H_02_v2 | 139370 | 141058 |
| ordered_PKA1H_02_v2 | 255572 | 264046 |
| ordered_PKA1H_02_v2 | 338351 | 346617 |
| ordered_PKA1H_02_v2 | 356653 | 362855 |
| ordered_PKA1H_02_v2 | 368899 | 369498 |
| ordered_PKA1H_02_v2 | 375745 | 376617 |
| ordered_PKA1H_02_v2 | 472537 | 474850 |
| ordered_PKA1H_02_v2 | 645363 | 646809 |
| ordered_PKA1H_02_v2 | 653240 | 654088 |
| ordered_PKA1H_02_v2 | 666569 | 668042 |
| ordered_PKA1H_02_v2 | 769028 | 769459 |
| ordered_PKA1H_02_v2 | 773156 | 774249 |
| ordered_PKA1H_02_v2 | 779195 | 787547 |
| ordered_PKA1H_03_v2 | 5888 | 10885 |
| ordered_PKA1H_03_v2 | 12108 | 16113 |
| ordered_PKA1H_03_v2 | 25778 | 29489 |
| ordered_PKA1H_03_v2 | 351307 | 361512 |
| ordered_PKA1H_03_v2 | 508470 | 508988 |
| ordered_PKA1H_03_v2 | 510845 | 514036 |
| ordered_PKA1H_03_v2 | 515369 | 517503 |
| ordered_PKA1H_03_v2 | 532228 | 535219 |
| ordered_PKA1H_03_v2 | 546585 | 549872 |
| ordered_PKA1H_03_v2 | 624308 | 625339 |
| ordered_PKA1H_03_v2 | 643466 | 651126 |
| ordered_PKA1H_03_v2 | 688074 | 690334 |
| ordered_PKA1H_03_v2 | 973653 | 975428 |
| ordered_PKA1H_03_v2 | 1008689 | 1011113 |
| ordered_PKA1H_03_v2 | 1025653 | 1037887 |
| ordered_PKA1H_03_v2 | 1042819 | 1044085 |
| ordered_PKA1H_04_v2 | 4708 | 20357 |
| ordered_PKA1H_04_v2 | 295995 | 301103 |
| ordered_PKA1H_04_v2 | 318609 | 319918 |
| ordered_PKA1H_04_v2 | 324026 | 325163 |
| ordered_PKA1H_04_v2 | 354500 | 363547 |
| ordered_PKA1H_04_v2 | 366018 | 366761 |
| ordered_PKA1H_04_v2 | 709222 | 716172 |
| ordered_PKA1H_04_v2 | 719475 | 720206 |
| ordered_PKA1H_04_v2 | 720946 | 722371 |
| ordered_PKA1H_04_v2 | 813556 | 816102 |
| ordered_PKA1H_04_v2 | 830003 | 831183 |
| ordered_PKA1H_04_v2 | 1069494 | 1081028 |
| ordered_PKA1H_04_v2 | 1125793 | 1126984 |
| ordered_PKA1H_05_v2 | 12252 | 13975 |
| ordered_PKA1H_05_v2 | 15998 | 18179 |
| ordered_PKA1H_05_v2 | 29489 | 31955 |
| ordered_PKA1H_05_v2 | 34970 | 36855 |
| ordered_PKA1H_05_v2 | 42878 | 44696 |
| ordered_PKA1H_05_v2 | 45463 | 46557 |
| ordered_PKA1H_05_v2 | 51375 | 52589 |
| ordered_PKA1H_05_v2 | 86989 | 90469 |
| ordered_PKA1H_05_v2 | 156841 | 158670 |
| ordered_PKA1H_05_v2 | 169143 | 171639 |
| ordered_PKA1H_05_v2 | 322092 | 352680 |
| ordered_PKA1H_05_v2 | 363454 | 373826 |
| ordered_PKA1H_05_v2 | 375663 | 377040 |
| ordered_PKA1H_05_v2 | 385744 | 386940 |
| ordered_PKA1H_05_v2 | 531047 | 532421 |
| ordered_PKA1H_05_v2 | 677294 | 679397 |
| ordered_PKA1H_06_v2 | 1983 | 10716 |
| ordered_PKA1H_06_v2 | 210286 | 212424 |
| ordered_PKA1H_06_v2 | 337904 | 340099 |
| ordered_PKA1H_06_v2 | 579786 | 580583 |
| ordered_PKA1H_06_v2 | 598394 | 599960 |
| ordered_PKA1H_06_v2 | 610442 | 613407 |
| ordered_PKA1H_06_v2 | 744484 | 746617 |
| ordered_PKA1H_06_v2 | 829217 | 830685 |
| ordered_PKA1H_06_v2 | 857514 | 858687 |
| ordered_PKA1H_06_v2 | 862714 | 864329 |
| ordered_PKA1H_06_v2 | 912838 | 915582 |
| ordered_PKA1H_06_v2 | 1035791 | 1037282 |
| ordered_PKA1H_06_v2 | 1059442 | 1062173 |
| ordered_PKA1H_06_v2 | 1067566 | 1069873 |
| ordered_PKA1H_06_v2 | 1074450 | 1076279 |
| ordered_PKA1H_07_v2 | 301123 | 304441 |
| ordered_PKA1H_07_v2 | 308609 | 310639 |
| ordered_PKA1H_07_v2 | 310681 | 321505 |
| ordered_PKA1H_07_v2 | 1056190 | 1064292 |
| ordered_PKA1H_07_v2 | 1083665 | 1084486 |
| ordered_PKA1H_07_v2 | 1108327 | 1119433 |
| ordered_PKA1H_07_v2 | 1162307 | 1163843 |
| ordered_PKA1H_07_v2 | 1203685 | 1204559 |
| ordered_PKA1H_07_v2 | 1227491 | 1229794 |
| ordered_PKA1H_07_v2 | 1505263 | 1513883 |
| ordered_PKA1H_07_v2 | 1515839 | 1516999 |
| ordered_PKA1H_08_v2 | 728 | 3043 |
| ordered_PKA1H_08_v2 | 39213 | 64655 |
| ordered_PKA1H_08_v2 | 227995 | 230691 |
| ordered_PKA1H_08_v2 | 307506 | 308870 |
| ordered_PKA1H_08_v2 | 368774 | 371423 |
| ordered_PKA1H_08_v2 | 379083 | 385376 |
| ordered_PKA1H_08_v2 | 394457 | 395892 |
| ordered_PKA1H_08_v2 | 613664 | 621505 |
| ordered_PKA1H_08_v2 | 633890 | 636389 |
| ordered_PKA1H_08_v2 | 643352 | 644492 |
| ordered_PKA1H_08_v2 | 726278 | 728601 |
| ordered_PKA1H_08_v2 | 798337 | 799775 |
| ordered_PKA1H_08_v2 | 811864 | 814328 |
| ordered_PKA1H_08_v2 | 815749 | 827798 |
| ordered_PKA1H_08_v2 | 924173 | 924586 |
| ordered_PKA1H_08_v2 | 937464 | 938132 |
| ordered_PKA1H_08_v2 | 998016 | 1015807 |
| ordered_PKA1H_08_v2 | 1018344 | 1020656 |
| ordered_PKA1H_08_v2 | 1097667 | 1100257 |
| ordered_PKA1H_08_v2 | 1183914 | 1199927 |
| ordered_PKA1H_08_v2 | 1201690 | 1204332 |
| ordered_PKA1H_08_v2 | 1365536 | 1380072 |
| ordered_PKA1H_08_v2 | 1381186 | 1384704 |
| ordered_PKA1H_08_v2 | 1438321 | 1439778 |
| ordered_PKA1H_08_v2 | 1440632 | 1445039 |
| ordered_PKA1H_08_v2 | 1460263 | 1461349 |
| ordered_PKA1H_08_v2 | 1702966 | 1704102 |
| ordered_PKA1H_08_v2 | 1708094 | 1709340 |
| ordered_PKA1H_08_v2 | 1721150 | 1726204 |
| ordered_PKA1H_08_v2 | 1730301 | 1750332 |
| ordered_PKA1H_08_v2 | 1901316 | 1903572 |
| ordered_PKA1H_08_v2 | 1920310 | 1921490 |
| ordered_PKA1H_09_v2 | 13604 | 14771 |
| ordered_PKA1H_09_v2 | 20490 | 21914 |
| ordered_PKA1H_09_v2 | 35482 | 37796 |
| ordered_PKA1H_09_v2 | 355002 | 356594 |
| ordered_PKA1H_09_v2 | 360572 | 362883 |
| ordered_PKA1H_09_v2 | 383187 | 384017 |
| ordered_PKA1H_09_v2 | 603267 | 604324 |
| ordered_PKA1H_09_v2 | 611621 | 612998 |
| ordered_PKA1H_09_v2 | 628463 | 630869 |
| ordered_PKA1H_09_v2 | 633597 | 636039 |
| ordered_PKA1H_09_v2 | 892671 | 899648 |
| ordered_PKA1H_09_v2 | 899687 | 903611 |
| ordered_PKA1H_09_v2 | 1118305 | 1119694 |
| ordered_PKA1H_09_v2 | 1313059 | 1325666 |
| ordered_PKA1H_09_v2 | 1413562 | 1425271 |
| ordered_PKA1H_09_v2 | 1857722 | 1867195 |
| ordered_PKA1H_09_v2 | 1937027 | 1945440 |
| ordered_PKA1H_09_v2 | 2159095 | 2162634 |
| ordered_PKA1H_09_v2 | 2171335 | 2173745 |
| ordered_PKA1H_09_v2 | 2182419 | 2183773 |
| ordered_PKA1H_10_v2 | 2089 | 3252 |
| ordered_PKA1H_10_v2 | 11891 | 14611 |
| ordered_PKA1H_10_v2 | 18311 | 27744 |
| ordered_PKA1H_10_v2 | 41937 | 44227 |
| ordered_PKA1H_10_v2 | 64320 | 66091 |
| ordered_PKA1H_10_v2 | 144343 | 145170 |
| ordered_PKA1H_10_v2 | 153125 | 161319 |
| ordered_PKA1H_10_v2 | 165874 | 167302 |
| ordered_PKA1H_10_v2 | 571870 | 573597 |
| ordered_PKA1H_10_v2 | 648203 | 677305 |
| ordered_PKA1H_10_v2 | 831636 | 844229 |
| ordered_PKA1H_10_v2 | 944814 | 946260 |
| ordered_PKA1H_10_v2 | 948339 | 949527 |
| ordered_PKA1H_10_v2 | 952518 | 966126 |
| ordered_PKA1H_10_v2 | 967628 | 968515 |
| ordered_PKA1H_10_v2 | 1346740 | 1367369 |
| ordered_PKA1H_10_v2 | 1489304 | 1493279 |
| ordered_PKA1H_10_v2 | 1500421 | 1509237 |
| ordered_PKA1H_10_v2 | 1513470 | 1516032 |
| ordered_PKA1H_11_v2 | 1241 | 2639 |
| ordered_PKA1H_11_v2 | 17041 | 20742 |
| ordered_PKA1H_11_v2 | 168955 | 189121 |
| ordered_PKA1H_11_v2 | 420715 | 433638 |
| ordered_PKA1H_11_v2 | 558625 | 560152 |
| ordered_PKA1H_11_v2 | 748803 | 757500 |
| ordered_PKA1H_11_v2 | 758875 | 760257 |
| ordered_PKA1H_11_v2 | 762156 | 775376 |
| ordered_PKA1H_11_v2 | 783762 | 784836 |
| ordered_PKA1H_11_v2 | 959772 | 963409 |
| ordered_PKA1H_11_v2 | 966702 | 968463 |
| ordered_PKA1H_11_v2 | 970136 | 972759 |
| ordered_PKA1H_11_v2 | 976194 | 982496 |
| ordered_PKA1H_11_v2 | 986804 | 987430 |
| ordered_PKA1H_11_v2 | 997353 | 998536 |
| ordered_PKA1H_11_v2 | 1189634 | 1192007 |
| ordered_PKA1H_11_v2 | 1398504 | 1400832 |
| ordered_PKA1H_11_v2 | 1416946 | 1417797 |
| ordered_PKA1H_11_v2 | 1757701 | 1775327 |
| ordered_PKA1H_11_v2 | 1784693 | 1798458 |
| ordered_PKA1H_11_v2 | 1901730 | 1904635 |
| ordered_PKA1H_11_v2 | 2090943 | 2092993 |
| ordered_PKA1H_11_v2 | 2094102 | 2096424 |
| ordered_PKA1H_11_v2 | 2110485 | 2111664 |
| ordered_PKA1H_11_v2 | 2187907 | 2194245 |
| ordered_PKA1H_11_v2 | 2197792 | 2199337 |
| ordered_PKA1H_11_v2 | 2322672 | 2324802 |
| ordered_PKA1H_11_v2 | 2345492 | 2347564 |
| ordered_PKA1H_11_v2 | 2352270 | 2359209 |
| ordered_PKA1H_12_v2 | 501162 | 508657 |
| ordered_PKA1H_12_v2 | 512274 | 514936 |
| ordered_PKA1H_12_v2 | 591554 | 595737 |
| ordered_PKA1H_12_v2 | 625131 | 626900 |
| ordered_PKA1H_12_v2 | 892602 | 894429 |
| ordered_PKA1H_12_v2 | 899036 | 901332 |
| ordered_PKA1H_12_v2 | 907676 | 908686 |
| ordered_PKA1H_12_v2 | 1192097 | 1204815 |
| ordered_PKA1H_12_v2 | 1954202 | 1955630 |
| ordered_PKA1H_12_v2 | 2119852 | 2122910 |
| ordered_PKA1H_12_v2 | 2126995 | 2140814 |
| ordered_PKA1H_12_v2 | 2262959 | 2275505 |
| ordered_PKA1H_12_v2 | 2434152 | 2459119 |
| ordered_PKA1H_12_v2 | 2492837 | 2495991 |
| ordered_PKA1H_12_v2 | 2496567 | 2497067 |
| ordered_PKA1H_12_v2 | 2581244 | 2582993 |
| ordered_PKA1H_12_v2 | 3139784 | 3140908 |
| ordered_PKA1H_12_v2 | 3146069 | 3154421 |
| ordered_PKA1H_12_v2 | 3159367 | 3160460 |
| ordered_PKA1H_13_v2 | 4195 | 16653 |
| ordered_PKA1H_13_v2 | 269586 | 270362 |
| ordered_PKA1H_13_v2 | 273108 | 290350 |
| ordered_PKA1H_13_v2 | 458100 | 460663 |
| ordered_PKA1H_13_v2 | 563950 | 566325 |
| ordered_PKA1H_13_v2 | 573318 | 579773 |
| ordered_PKA1H_13_v2 | 581565 | 586107 |
| ordered_PKA1H_13_v2 | 675092 | 676986 |
| ordered_PKA1H_13_v2 | 679868 | 682474 |
| ordered_PKA1H_13_v2 | 867123 | 869056 |
| ordered_PKA1H_13_v2 | 872099 | 873031 |
| ordered_PKA1H_13_v2 | 1029999 | 1032499 |
| ordered_PKA1H_13_v2 | 1139820 | 1147469 |
| ordered_PKA1H_13_v2 | 1152110 | 1154455 |
| ordered_PKA1H_13_v2 | 1157003 | 1159269 |
| ordered_PKA1H_13_v2 | 1176038 | 1177396 |
| ordered_PKA1H_13_v2 | 1179083 | 1181461 |
| ordered_PKA1H_13_v2 | 1188085 | 1191664 |
| ordered_PKA1H_13_v2 | 1248305 | 1250700 |
| ordered_PKA1H_13_v2 | 1253625 | 1271814 |
| ordered_PKA1H_13_v2 | 1401057 | 1421456 |
| ordered_PKA1H_13_v2 | 1425818 | 1428490 |
| ordered_PKA1H_13_v2 | 1821724 | 1830971 |
| ordered_PKA1H_13_v2 | 1994575 | 2007150 |
| ordered_PKA1H_13_v2 | 2012832 | 2014451 |
| ordered_PKA1H_13_v2 | 2564854 | 2566712 |
| ordered_PKA1H_13_v2 | 2586643 | 2587377 |
| ordered_PKA1H_13_v2 | 2592861 | 2594211 |
| ordered_PKA1H_14_v2 | 654 | 3160 |
| ordered_PKA1H_14_v2 | 17151 | 18884 |
| ordered_PKA1H_14_v2 | 332198 | 344025 |
| ordered_PKA1H_14_v2 | 543851 | 545047 |
| ordered_PKA1H_14_v2 | 558278 | 560698 |
| ordered_PKA1H_14_v2 | 565738 | 566898 |
| ordered_PKA1H_14_v2 | 709966 | 712453 |
| ordered_PKA1H_14_v2 | 854231 | 855844 |
| ordered_PKA1H_14_v2 | 1128992 | 1135609 |
| ordered_PKA1H_14_v2 | 1197032 | 1207819 |
| ordered_PKA1H_14_v2 | 1409699 | 1422988 |
| ordered_PKA1H_14_v2 | 1457860 | 1458672 |
| ordered_PKA1H_14_v2 | 1460906 | 1463550 |
| ordered_PKA1H_14_v2 | 1945008 | 1947564 |
| ordered_PKA1H_14_v2 | 2232745 | 2234976 |
| ordered_PKA1H_14_v2 | 2237197 | 2238968 |
| ordered_PKA1H_14_v2 | 2249798 | 2250837 |
| ordered_PKA1H_14_v2 | 2405969 | 2407501 |
| ordered_PKA1H_14_v2 | 2414607 | 2423999 |
| ordered_PKA1H_14_v2 | 2438995 | 2440270 |
| ordered_PKA1H_14_v2 | 2623485 | 2627281 |
| ordered_PKA1H_14_v2 | 2631052 | 2632221 |
| ordered_PKA1H_14_v2 | 2644281 | 2654242 |
| ordered_PKA1H_14_v2 | 2658716 | 2661101 |
| ordered_PKA1H_14_v2 | 2752736 | 2754356 |
| ordered_PKA1H_14_v2 | 2765828 | 2767444 |
| ordered_PKA1H_14_v2 | 2865860 | 2866741 |
| ordered_PKA1H_14_v2 | 2871799 | 2876262 |
| ordered_PKA1H_14_v2 | 2885951 | 2895816 |
| ordered_PKA1H_14_v2 | 3091657 | 3094121 |
| ordered_PKA1H_14_v2 | 3097172 | 3099681 |
| ordered_PKA1H_14_v2 | 3239908 | 3241773 |
| ordered_PKA1H_14_v2 | 3257371 | 3259715 |
| ordered_PKA1H_14_v2 | 3262939 | 3264448 |
| ordered_PKA1H_02_v2 | 336890 | 338169 |
| ordered_PKA1H_12_v2 | 876149 | 877508 |
| ordered_PKA1H_12_v2 | 2125953 | 2126932 |
| ordered_PKA1H_01_v2 | 0 | 31756 |
| ordered_PKA1H_01_v2 | 851582 | 880986 |
| ordered_PKA1H_02_v2 | 0 | 39903 |
| ordered_PKA1H_02_v2 | 717562 | 728134 |
| ordered_PKA1H_03_v2 | 0 | 40085 |
| ordered_PKA1H_03_v2 | 984128 | 1030967 |
| ordered_PKA1H_04_v2 | 0 | 45677 |
| ordered_PKA1H_04_v2 | 1111994 | 1125182 |
| ordered_PKA1H_05_v2 | 0 | 40025 |
| ordered_PKA1H_05_v2 | 735661 | 741125 |
| ordered_PKA1H_06_v2 | 0 | 19789 |
| ordered_PKA1H_06_v2 | 1045508 | 1063895 |
| ordered_PKA1H_07_v2 | 0 | 9141 |
| ordered_PKA1H_07_v2 | 1485805 | 1522165 |
| ordered_PKA1H_08_v2 | 0 | 17009 |
| ordered_PKA1H_08_v2 | 1874898 | 1888040 |
| ordered_PKA1H_09_v2 | 0 | 56545 |
| ordered_PKA1H_09_v2 | 2085215 | 2162603 |
| ordered_PKA1H_10_v2 | 0 | 69857 |
| ordered_PKA1H_10_v2 | 1439673 | 1481301 |
| ordered_PKA1H_11_v2 | 0 | 29067 |
| ordered_PKA1H_11_v2 | 2317969 | 2356946 |
| ordered_PKA1H_12_v2 | 0 | 38750 |
| ordered_PKA1H_12_v2 | 3129934 | 3155749 |
| ordered_PKA1H_13_v2 | 0 | 24663 |
| ordered_PKA1H_13_v2 | 2519037 | 2563645 |
| ordered_PKA1H_14_v2 | 0 | 46265 |
| ordered_PKA1H_14_v2 | 3204808 | 3238094 |

**Supplementary Table B.** Hypervariable genomic regions masked from analysis. This includes sub telomeric regions, and *SICAvar* and *Kir* genes.

| Filtering method | SNP count | *Mn*-*Mn* median IBD |
| --- | --- | --- |
| Original - described in methods | 340787 | 0.4642 |
| Genotypic missingness <0.05 | 286373 | 0.44643 |
| Genotypic missingness <0.01 | 215935 | 0.46354 |
| Fst <0.9 & genotypic missingness <0.01 | 188557 | 0.34635 (*Mf*-*Mf* = 0.01748) |
| Fst <0.75 & genotypic missingness <0.01 | 167405 | 0.28292 (*Mf*-*Mf* = 0.01101) |
| Removal of SNPs that segregate to a cluster (& present in at least >50% of that cluster) | 331927 | 0.41664 |
| Separate MAF filters (>0.05) for each cluster | 602079 | 0.41690 |

**Supplementary Table C.** Limited variation of median *Mn-Mn* IBD values when trialling different filtering methods that results in different SNP counts prior to IBD calculations with hmmIBD.

| Sample | Windows (n) | Tertile | Cluster | State | District |
| --- | --- | --- | --- | --- | --- |
| PK_SB_DNA_028 | 37 | High | Mf | Sabah | Papar |
| ERR985380 | 34 | High | Mf | Sarawak | Betong |
| ERR985379 | 33 | High | Mf | Sarawak | Betong |
| ERR985373 | 32 | High | Mf | Sarawak | Betong |
| ERR985381 | 32 | High | Mf | Sarawak | Betong |
| ERR985372 | 31 | High | Mf | Sarawak | Betong |
| ERR985384 | 31 | High | Mf | Sarawak | Betong |
| ERR985377 | 29 | High | Mf | Sarawak | Betong |
| ERR985378 | 29 | High | Mf | Sarawak | Betong |
| ERR985375 | 24 | High | Mf | Sarawak | Betong |
| ERR274221 | 23 | High | Mf | Sarawak | Sarikei |
| ERR985374 | 22 | High | Mf | Sarawak | Betong |
| PK_SB_DNA_053 | 13 | High | Mf | Sabah | Kudat |
| ERR274222 | 12 | High | Mf | Sarawak | Sarikei |
| PK_SB_DNA_087 | 10 | High | Mf | Sabah | Pitas |
| ERR985406 | 9 | High | Mf | Sarawak | Kapit |
| ERR985382 | 8 | High | Mf | Sarawak | Betong |
| ERR985383 | 8 | High | Mf | Sarawak | Betong |
| PK_SB_DNA_003 | 8 | High | Mf | Sabah | Kudat |
| PK_SB_DNA_077 | 8 | High | Mf | Sabah | Kudat |
| ERR985401 | 7 | High | Mf | Sarawak | Kapit |
| PK_SB_DNA_005 | 7 | High | Mf | Sabah | Kudat |
| PK_SB_DNA_031 | 7 | High | Mf | Sabah | Kudat |
| PK_SB_DNA_049 | 7 | High | Mf | Sabah | Pitas |
| PK_SB_DNA_072 | 7 | High | Mf | Sabah | Kota Marudu |
| ERR366426 | 6 | High | Mf | Sarawak | Sarikei |
| ERR985388 | 6 | High | Mf | Sarawak | Kapit |
| ERR985390 | 6 | High | Mf | Sarawak | Kapit |
| ERR985393 | 6 | High | Mf | Sarawak | Kapit |
| ERR985394 | 6 | High | Mf | Sarawak | Kapit |
| ERR985407 | 6 | High | Mf | Sarawak | Kapit |
| PK_SB_DNA_002 | 6 | High | Mf | Sabah | Kota Kinabalu |
| PK_SB_DNA_013 | 6 | High | Mf | Sabah | Kudat |
| PK_SB_DNA_047 | 6 | High | Mf | Sabah | Kota Marudu |
| PK_SB_DNA_090 | 6 | High | Mf | Sabah | Kota Marudu |
| ERR985385 | 5 | Medium | Mf | Sarawak | Kapit |
| ERR985391 | 5 | Medium | Mf | Sarawak | Kapit |
| ERR985398 | 5 | High | Mf | Sarawak | Kapit |
| ERR985409 | 5 | High | Mf | Sarawak | Kapit |
| PK_SB_DNA_001 | 5 | High | Mf | Sabah | Kota Belud |
| PK_SB_DNA_009 | 5 | High | Mf | Sabah | Tuaran |
| PK_SB_DNA_023 | 5 | High | Mf | Sabah | Kudat |
| PK_SB_DNA_035 | 5 | High | Mf | Sabah | Kota Marudu |
| PK_SB_DNA_057 | 5 | High | Mf | Sabah | Kota Marudu |
| PK_SB_DNA_067 | 5 | High | Mf | Sabah | Kota Marudu |
| PK_SB_DNA_068 | 5 | High | Mf | Sabah | Kota Marudu |
| ERR3374032 | 4 | Medium | Peninsular | Kelantan | Gua_Musang |
| ERR3374040 | 4 | Medium | Peninsular | Pahang | Kuala Lipis |
| ERR3374057 | 4 | Medium | Peninsular | Perak | Taiping |
| ERR985376 | 4 | Medium | Mf | Sarawak | Betong |
| ERR985389 | 4 | Medium | Mf | Sarawak | Kapit |
| ERR985400 | 4 | Medium | Mf | Sarawak | Kapit |
| ERR985403 | 4 | Medium | Mf | Sarawak | Kapit |
| ERR985408 | 4 | Medium | Mf | Sarawak | Kapit |
| PK_SB_DNA_008 | 4 | Medium | Mf | Sabah | Kota Kinabalu |
| PK_SB_DNA_010 | 4 | Medium | Mf | Sabah | Kudat |
| PK_SB_DNA_021 | 4 | Medium | Mf | Sabah | Penampang |
| PK_SB_DNA_029 | 4 | Medium | Mf | Sabah | Kota Marudu |
| PK_SB_DNA_030 | 4 | Medium | Mn | Sabah | Kudat |
| PK_SB_DNA_034 | 4 | Medium | Mf | Sabah | Pitas |
| PK_SB_DNA_040 | 4 | Medium | Mf | Sabah | Pitas |
| PK_SB_DNA_044 | 4 | Medium | Mf | Sabah | Kota Marudu |
| PK_SB_DNA_045 | 4 | Medium | Mf | Sabah | Kota Marudu |
| PK_SB_DNA_052 | 4 | Medium | Mf | Sabah | Pitas |
| PK_SB_DNA_081 | 4 | Medium | Mf | Sabah | Kudat |
| PK_SB_DNA_086 | 4 | Medium | Mf | Sabah | Kota Marudu |
| ERR3374033 | 3 | Medium | Peninsular | Kelantan | Gua Musang |
| ERR3374039 | 3 | Medium | Peninsular | Pahang | Kuala Lipis |
| ERR3374042 | 3 | Medium | Peninsular | Pahang | Kuala Lipis |
| ERR3374045 | 3 | Medium | Peninsular | Pahang | Kuala Lipis |
| ERR3374048 | 3 | Medium | Peninsular | Pahang | Kuala Lipis |
| ERR3374050 | 3 | Medium | Peninsular | Pahang | Kuala Lipis |
| ERR3374054 | 3 | Medium | Peninsular | Perak | Sungai Siput |
| ERR985387 | 3 | Medium | Mf | Sarawak | Kapit |
| ERR985392 | 3 | Medium | Mf | Sarawak | Kapit |
| ERR985404 | 3 | Medium | Mf | Sarawak | Kapit |
| ERR985412 | 3 | Medium | Mn | Sarawak | Kapit |
| PK_SB_DNA_014 | 3 | Medium | Mf | Sabah | Ranau |
| PK_SB_DNA_015 | 3 | Medium | Mf | Sabah | Kudat |
| PK_SB_DNA_036 | 3 | Medium | Mf | Sabah | Kota Marudu |
| PK_SB_DNA_042 | 3 | Medium | Mn | Sabah | Ranau |
| PK_SB_DNA_046 | 3 | Medium | Mf | Sabah | Kota Marudu |
| PK_SB_DNA_063 | 3 | Medium | Mn | Sabah | Ranau |
| PK_SB_DNA_069 | 3 | Medium | Mf | Sabah | Kota Marudu |
| PK_SB_DNA_070 | 3 | Medium | Mf | Sabah | Kota Marudu |
| PK_SB_DNA_073 | 3 | Medium | Mf | Sabah | Kudat |
| PK_SB_DNA_074 | 3 | Medium | Mf | Sabah | Kota Marudu |
| PK_SB_DNA_088 | 3 | Medium | Mf | Sabah | Kota Marudu |
| ERR2214840 | 2 | Low | Mn | Sarawak | Kapit |
| ERR2214847 | 2 | Low | Mn | Sarawak | Kapit |
| ERR2214852 | 2 | Low | Mn | Sarawak | Kapit |
| ERR2214857 | 2 | Low | Mn | Sarawak | Kapit |
| ERR3374031 | 2 | Low | Peninsular | Kelantan | Gua Musang |
| ERR3374035 | 2 | Low | Peninsular | Pahang | Kuala Lipis |
| ERR3374037 | 2 | Low | Peninsular | Pahang | Kuala Lipis |
| ERR3374047 | 2 | Low | Peninsular | Pahang | Kuala Lipis |
| ERR3374049 | 2 | Low | Peninsular | Pahang | Kuala Lipis |
| ERR3374051 | 2 | Low | Peninsular | Perak | Sungai Siput |
| ERR3374055 | 2 | Low | Peninsular | Pahang | Temerloh |
| ERR3374056 | 2 | Low | Peninsular | Pahang | Temerloh |
| ERR3374058 | 2 | Low | Peninsular | Perak | Taiping |
| ERR985399 | 2 | Low | Mf | Sarawak | Kapit |
| ERR985402 | 2 | Low | Mf | Sarawak | Kapit |
| PK_SB_DNA_038 | 2 | Low | Mf | Sabah | Kota Marudu |
| PK_SB_DNA_054 | 2 | Low | Mf | Sabah | Kudat |
| PK_SB_DNA_082 | 2 | Low | Mf | Sabah | Kudat |
| PK_SB_DNA_089 | 2 | Low | Mf | Sabah | Beluran |
| PK_SB_DNA_093 | 2 | Low | Mn | Sabah | Kota Marudu |
| PK_SB_DNA_094 | 2 | Low | Mf | Sabah | Kota Marudu |
| SRR2225571 | 2 | Low | Peninsular | Peninsular | Clinic |
| SRR2225573 | 2 | Low | Peninsular | Peninsular | Clinic |
| ERR2214837 | 1 | Low | Mn | Sarawak | Kapit |
| ERR2214838 | 1 | Low | Mn | Sarawak | Kapit |
| ERR2214841 | 1 | Low | Mn | Sarawak | Kapit |
| ERR2214845 | 1 | Low | Mn | Sarawak | Kapit |
| ERR2214848 | 1 | Low | Mn | Sarawak | Kapit |
| ERR2214853 | 1 | Low | Mn | Sarawak | Kapit |
| ERR274224 | 1 | Low | Mn | Sarawak | Sarikei |
| ERR274225 | 1 | Low | Mn | Sarawak | Sarikei |
| ERR3374034 | 1 | Low | Peninsular | Kelantan | Gua Musang |
| ERR3374036 | 1 | Low | Peninsular | Pahang | Kuala Lipis |
| ERR3374038 | 1 | Low | Peninsular | Pahang | Kuala Lipis |
| ERR3374043 | 1 | Low | Peninsular | Pahang | Kuala Lipis |
| ERR3374044 | 1 | Low | Peninsular | Pahang | Kuala Lipis |
| ERR3374052 | 1 | Low | Peninsular | Perak | Sungai Siput |
| ERR3374053 | 1 | Low | Peninsular | Perak | Sungai Siput |
| ERR985411 | 1 | Low | Mn | Sarawak | Betong |
| ERR985413 | 1 | Low | Mn | Sarawak | Kapit |
| ERR985418 | 1 | Low | Mn | Sarawak | Kapit |
| ERR985419 | 1 | Low | Mn | Sarawak | Kapit |
| PK_SB_DNA_011 | 1 | Low | Mn | Sabah | Papar |
| PK_SB_DNA_043 | 1 | Low | Mn | Sabah | Kota Marudu |
| PK_SB_DNA_092 | 1 | Low | Mn | Sabah | Kota Marudu |

**Supplementary Table D.** Number of introgressed windows identified for each sample, and the associated cluster, state and district information.

| Comparison | Estimate | Standard Error | *t-*value | *p*-value |
| --- | --- | --- | --- | --- |
| *Anopheles* Leucosphyrus Complex-mosquito distribution & genomic cluster | 0.74 | 0.95 | 0.78 | 0.44 |
| Percentage tree cover & genomic cluster | 0.00093 | 0.002 | 0.386 | 0.70 |
| Perimeter-area ratio for tree cover & genomic cluster | -1.12 | 0.89 | -1.26 | 0.22 |
| Distribution of *Anopheles* Leucosphyrus Complex-mosquito & degree of introgression | -1.29 | 1.60 | -0.81 | 0.42 |
| Percentage tree cover & degree of introgression | 0.004 | 0.01 |  | 0.74 |
| Perimeter-area ratio for tree cover & degree of introgression | 10.03 | 8.81 |  | 0.26 |

**Supplementary Table E.** Results of linear and ordinal regression analyses using optimal models, as determined by AIC comparisons.

| Comparison | *ꭓ^2^* | df | *p*-value | r^2^ |
| --- | --- | --- | --- | --- |
| Percentage tree cover and presence of window 859 | 8.17 | 1 | < 0.01 | 0.71 |
| Perimeter-area ratio for tree cover and presence of window 1236 | 6 | 1 | 0.02 | 0.69 |

**Supplementary Table F.** Results of logistic regression analyses (significant outcomes) using optimal models, as determined by AIC comparisons, and used for determining associations between presence/absence of windows and environmental factors.

| Window | Chromosome | Start position | End position |
| --- | --- | --- | --- |
| 822 | 08 | 910000 | 919999 |
| 854 | 08 | 1230000 | 1239999 |
| 855 | 08 | 1240000 | 1249999 |
| 856 | 08 | 1250000 | 1259999 |
| 858 | 08 | 1270000 | 1279999 |
| 909 | 08 | 1780000 | 1789999 |
| 1196 | 10 | 520000 | 529999 |
| 1216 | 10 | 520000 | 529999 |

**Supplementary Table G.** Table of introgressed windows and their associated genomic positions for overlapping windows found in the two outlier isolates from Sabah (PK_SB_DNA_028 & PK_SB_DNA_053) and several Sarawak isolates.

| **CHR** | **SNP** | **POS** | **NMISS** | **F_ST_** |
| --- | --- | --- | --- | --- |
| 01 | A:G | 640680 | 70 | 0.538488 |
| 02 | C:A | 58980 | 75 | 0.516361 |
| 02 | T:C | 58987 | 77 | 0.576825 |
| 02 | T:C | 413060 | 78 | 0.530008 |
| 03 | G:A | 982721 | 62 | 0.606347 |
| 04 | G:C | 611739 | 80 | 0.542445 |
| 04 | T:C | 1021954 | 81 | 0.553455 |
| 04 | A:G | 1022135 | 81 | 0.684487 |
| 04 | G:A | 1023086 | 81 | 0.526905 |
| 04 | C:A | 1023688 | 79 | 0.678805 |
| 04 | T:A | 1023919 | 80 | 0.747473 |
| 04 | G:T | 1023971 | 81 | 0.684487 |
| 04 | G:A | 1023981 | 81 | 0.684487 |
| 04 | A:G | 1024302 | 75 | 0.666785 |
| 05 | A:G | 185185 | 80 | 0.536652 |
| 06 | G:C | 693645 | 81 | 0.555408 |
| 06 | T:C | 693780 | 81 | 0.738752 |
| 06 | C:T | 693845 | 80 | 0.654148 |
| 06 | T:C | 694548 | 81 | 0.602859 |
| 06 | G:T | 695672 | 79 | 0.733691 |
| 06 | C:G | 696277 | 81 | 0.643145 |
| 07 | A:G | 29979 | 75 | 0.515779 |
| 07 | G:A | 29980 | 75 | 0.515779 |
| 07 | G:A | 472868 | 79 | 0.534319 |
| 07 | G:A | 472888 | 79 | 0.554396 |
| 07 | G:C | 1243315 | 81 | 0.921223 |
| 07 | G:T | 1243339 | 81 | 0.921223 |
| 07 | T:G | 1243417 | 81 | 0.921223 |
| 07 | G:A | 1474592 | 76 | 0.551749 |
| 08 | A:C | 430385 | 59 | 0.682104 |
| 08 | A:T | 502644 | 77 | 0.570506 |
| 08 | C:T | 502691 | 73 | 0.631982 |
| 08 | G:T | 502739 | 75 | 0.63861 |
| 08 | T:G | 502957 | 71 | 0.625094 |
| 08 | A:G | 503004 | 72 | 0.628571 |
| 08 | T:A | 503070 | 76 | 0.585492 |
| 08 | T:C | 574975 | 80 | 0.697155 |
| 08 | A:T | 948013 | 81 | 0.512422 |
| 08 | G:A | 948893 | 81 | 0.527866 |
| 08 | C:G | 949304 | 81 | 0.512422 |
| 09 | C:T | 742539 | 54 | 0.526938 |
| 10 | C:* | 380211 | 52 | 0.518846 |
| 11 | T:C | 1425835 | 81 | 0.556235 |
| 11 | G:A | 1426186 | 80 | 0.570615 |
| 11 | G:T | 1427109 | 81 | 0.615943 |
| 11 | C:A | 1427114 | 81 | 0.615943 |
| 11 | A:G | 1449047 | 81 | 0.585385 |
| 11 | G:C | 1449048 | 81 | 0.585385 |
| 11 | G:C | 1449303 | 81 | 0.585385 |
| 11 | G:A | 1449959 | 81 | 0.671754 |
| 11 | G:C | 1450079 | 65 | 0.50685 |
| 11 | C:T | 1450118 | 80 | 0.668552 |
| 11 | C:T | 1451730 | 80 | 0.651105 |
| 11 | T:C | 1451731 | 80 | 0.651105 |
| 11 | A:G | 1451739 | 80 | 0.665502 |
| 11 | A:G | 1451750 | 79 | 0.6218 |
| 11 | G:T | 1451781 | 80 | 0.668552 |
| 11 | C:A | 1451806 | 81 | 0.671754 |
| 11 | G:C | 1451809 | 81 | 0.671754 |
| 11 | A:T | 1451810 | 81 | 0.671754 |
| 11 | G:T | 1451814 | 81 | 0.671754 |
| 11 | C:T | 1451921 | 81 | 0.671754 |
| 11 | A:T | 1451949 | 81 | 0.671754 |
| 11 | A:G | 1452439 | 78 | 0.539502 |
| 11 | C:T | 1832616 | 64 | 0.53053 |
| 12 | G:A | 843057 | 58 | 0.647144 |
| 12 | C:T | 1242081 | 80 | 0.501948 |
| 12 | G:A | 1242748 | 80 | 0.584409 |
| 12 | T:C | 2084429 | 60 | 0.539902 |
| 12 | C:G | 2094330 | 72 | 0.61139 |
| 12 | C:T | 2095080 | 79 | 0.611234 |
| 12 | C:A | 2096035 | 80 | 0.700454 |
| 12 | A:G | 2104236 | 74 | 0.787706 |
| 12 | G:A | 2500705 | 81 | 0.553455 |
| 13 | G:C | 55312 | 78 | 0.85994 |
| 13 | C:T | 1959231 | 61 | 0.537083 |
| 13 | T:C | 2165202 | 69 | 0.789097 |
| 13 | T:G | 2165740 | 80 | 0.628963 |
| 13 | T:* | 2165740 | 80 | 0.628963 |
| 13 | C:A | 2165741 | 80 | 0.628963 |
| 13 | C:* | 2165741 | 80 | 0.628963 |
| 13 | T:C | 2165805 | 81 | 0.517352 |
| 14 | G:C | 1631601 | 78 | 0.553329 |

**Supplementary Table H.** List of SNPs above 0.5 threshold from the F_ST_ analysis comparing Sarawak and Sabah isolates from the *Mf* cluster.

| **Gene ID** | **Genomic Location(s)** | **Product Description** | **Chromosome** |
| --- | --- | --- | --- |
| PKA1H_040027900 | LT727651: 1,022,201 - 1,027,227 (+) | conserved Plasmodium protein, unknown function | 4 |
| PKA1H_070033400 | LT727654: 1,242,436 - 1,247,902 (+) | high molecular weight rhoptry protein 2, putative | 7 |
| PKA1H_080016100 | LT727655: 502,561 - 503,088 (-) | conserved Plasmodium protein, unknown function | 8 |
| PKA1H_080026000 | LT727655: 940,469 - 950,653 (+) | oocyst capsule protein, putative | 8 |
| PKA1H_110035900 | LT727658: 1,425,595 - 1,426,125 (-) | hypothetical protein, conserved | 11 |
| PKA1H_110036000 | LT727658: 1,428,564 - 1,429,568 (+) | conserved Plasmodium protein, unknown function | 11 |
| PKA1H_110036100 | LT727658: 1,430,328 - 1,431,026 (-) | splicing factor 3A subunit 2, putative | 11 |
| PKA1H_110036200 | LT727658: 1,432,477 - 1,439,337 (+) | conserved Plasmodium protein, unknown function | 11 |
| PKA1H_110036300 | LT727658: 1,440,360 - 1,442,733 (+) | conserved Plasmodium protein, unknown function | 11 |
| PKA1H_110036400 | LT727658: 1,442,897 - 1,445,143 (-) | conserved Plasmodium protein, unknown function | 11 |
| PKA1H_110036500 | LT727658: 1,445,994 - 1,450,007 (+) | acyl-CoA synthetase, putative | 11 |
| PKA1H_110036600 | LT727658: 1,451,470 - 1,452,471 (+) | conserved Plasmodium protein, unknown function | 11 |
| PKA1H_120050800 | LT727659: 2,083,200 - 2,084,966 (+) | conserved Plasmodium protein, unknown function | 12 |
| PKA1H_120050900 | LT727659: 2,087,277 - 2,087,730 (-) | conserved Plasmodium protein, unknown function | 12 |
| PKA1H_120051000 | LT727659: 2,091,475 - 2,092,002 (+) | early transcribed membrane protein | 12 |
| PKA1H_120051100 | LT727659: 2,094,098 - 2,096,799 (-) | Plasmodium exported protein, unknown function | 12 |
| PKA1H_120051200 | LT727659: 2,099,000 - 2,099,912 (-) | Plasmodium exported protein, unknown function | 12 |
| PKA1H_120051300 | LT727659: 2,104,172 - 2,105,354 (+) | DNAJ like protein, putative | 12 |
| PKA1H_120051400 | LT727659: 2,107,943 - 2,110,430 (-) | Plasmodium exported protein, unknown function | 12 |
| PKA1H_120059100 | LT727659: 2,464,712 - 2,466,064 (+) | conserved Plasmodium protein, unknown function | 12 |
| PKA1H_130005900 | LT727660: 54,290 - 55,452 (+) | tryptophan-rich antigen, putative | 13 |
| PKA1H_130052600 | LT727660: 2,164,214 - 2,166,124 (-) | rhoptry-associated protein 1, putative | 13 |
| PKA1H_140043200 | LT727661: 1,629,491 - 1,632,232 (-) | transporter, putative | 14 |

**Supplementary Table I.** List of genes overlapping SNPs identified above 0.5 threshold from the F_ST_ analysis comparing Sarawak and Sabah isolates from the *Mf* cluster.
