# Supplementary material for: Genomic epidemiology of Plasmodium knowlesi reveals putative genetic drivers of adaptation in Malaysia
Source: PLoS Negl Trop Dis. 2025 Mar 12;19(3):e0012885. doi: 10.1371/journal.pntd.0012885 (PMC11932472; doi:10.1371/journal.pntd.0012885)
Supplement: Attachment — Submitted filename: Reviewer_comments_281024_mg_jw.docx [file pntd.0012885.s003.docx]

| **Reviewer** | **Comment Type** | **Manuscript Section** | **Comment** |  |
| --- | --- | --- | --- | --- |
| Reviewer #1 | Major | Methods | Please see ‘general comments.’ | NA |
| Reviewer #2 | Minor | Methods | No response. | NA |
| Reviewer #3 | Major | Methods | 1. **Re: sample collection.** The authors should indicate whether the samples included in the study were single-species Pk infections. Were they tested for Pf, Pv, etc.? If not, why not? If so, how were co-infected samples dealt with? Were they excluded for ease of analysis? Did the laboratory or bioinformatic methods selectively amplify Pk vs other Plasmodium DNA?  2. **Re: variant detection pipeline.** The authors indicate that they used a modified version of a previously described workflow, but do not comment on what those modifications were. It appears that they largely followed GATK Best Practices, with a few notable deviations that are not explained:  a. Why was GATK 3.8 used instead of GATK 4, which has been available since 2018?  b. How was the “conservative set of high-quality variants” generated? What metrics were used to designate them?  c. The base recalibration and indel realignment method is unlike any I have seen before and does not follow GATK’s best practices guidelines, which recommend using hard filtering in cases where there is no available variant database. What was the justification for using the chosen method rather than simply looking at the distributions of quality metrics (e.g. FS, QD, MQ) in the entire variant callset and choosing conservative cutoffs?  d. The decision to use both bcftools mpileup and GATK HaplotypeCaller is not explained. Presumably, the authors determined that variants identified by both methods were higher quality, but this should be spelled out as it is not a standard method.  3. **Re: population structure.**  a. The chosen thresholds for IBD plots are very confusing, both in terms of the written description and in terms of the plots, which do not match the description. My best interpretation of the description is that the authors started with 95%, then halved it to 42.5%, then halved it again, which would be 21.25%. However, the actual cutoffs used are different, with no explanation as to why. In addition, the choice to do this at all is confusing. Why not just use standard cutoffs, e.g. 95%, 90%, 50%, 25%, 10%, etc.? The authors may have an intuitive sense of what IBD of 11.8% means biologically, but I would suspect they are in the minority if so.  b. CV error values from ADMIXTURE should be given, if only in a supplementary graph. Specifically, how different are the values for 3 populations vs. 2 or 4? The results are extremely convincing that there are 3 populations, but including these values would add additional support.  4. **Re: characterizing subpopulations.** The authors indicate that they controlled for population structure by subsetting samples, but they do not explain how this subsetting occurred. What were the thresholds? What was the subsetting scheme (i.e. was it proportional? random? data-driven?)? | 1. The samples were single species, Pk infections (confirmed through PCR), and we have now amended the methods to clarify this. No sWGA method was used on this dataset.  2a. The re-engineering of GATK3 to version 4 by Broad was to enhance speed, resource efficiency and scalability, with very small differences on how GATK3 and GATK4 work internally. However, we chose to use GATK 3.8 for the simple reason that we wanted to use the indel realignment step prior to haplotype caller (which we recognise also has a realignment mechanism) in a streamlined manner (single software version – 3.8). According to Broad, subtle differences observed (between indel realignment and not using it) may become more substantial when dealing with low quality data. Although our data quality is high in this dataset, we designed the pipeline within the broader scope of our program in mind, which may include working with lower quality datasets produced by partners in the future.  2b & 2c. For the GATK component, we follow the best practice workflow of GATK4, but with the addition of indel realignment and using 3.8. Indel realignment is from the GATK3 best practice workflow and base recalibration is in both the GATK 3 & 4 best practice workflows. For recalibration (BQSR), Broad recommend that you take a [bootstrap approach](https://gatk.broadinstitute.org/hc/en-us/articles/360035890531-Base-Quality-Score-Recalibration-BQSR) for non-model organisms (when there is no reference data). In this approach, you use hard filtering, as you suggest, creating a set of variants that can then be passed back into the recalibration step. Once the next iteration of the pipeline is complete, we perform hard filtering again. We have now clarified this in text and provided a reference for the bootstrapping approach briefly described by Broad.  2d. The motivation behind the consensus approach is that different variant callers use distinct algorithms and heuristics, typically biasing them towards either sensitivity or precision, and combining their results could improve accuracy and reduce false positives (if taking a consensus and not aggregating). Although still uncommon, the approach has been explored for variant calling ([Example 1](https://www.nature.com/articles/s41598-020-69772-8.pdf), [example 2,](https://www.nature.com/articles/s41598-023-34925-y.pdf) [example 3](https://academic.oup.com/bioinformatics/article/40/2/btae066/7609103#441550238)), and it has become increasingly common for other common algorithms and analyses (e.g. differential expression and differential abundance analyses). We have added a brief statement in the methods to justify.  3a. There was no biological reasoning it is just the default setting from our script to take half of 95 and half repeatedly. However, in hindsight, we agree that it is not intuitive (and we have also deviated from the method described) so we have changed the thresholds to those suggested, in alignment with many other manuscripts. We appreciate the comment, because it also led to an additional observation – an Mf sample (with high rates of introgression) maintaining connectivity with Mn at 10%, despite no connectivity with other Mf samples at this threshold.  3b. We have added a plot of the CV error across K=6 to the supplementary material.  4. The subsets were cluster specific (Mf and Mn), and we used NJT and ADMIXTURE to assign the samples to the appropriate clusters for this analysis. We have now clarified this in the methods. |
| Reviewer #1 | Major | Results | Please see ‘general comments.’ | NA |
| Reviewer #2 | Minor | Results | No response. | NA |
| Reviewer #3 | Major | Results | 1. **Re: coverage.**  a. 30.27% of reads mapped to the Pk reference genome? What were the rest? Human? Macaque? Other Plasmodium coinfections?  b. How evenly distributed was coverage across the chromosomes? How variable was coverage between samples? The overall average is given, but not a range.  2. **Re: clonality.**  a. Fws is a sufficient metric for distinguishing monoclonal from polyclonal infections, however it is fairly arbitrary and less informative than COI/MOI. Particularly given that Fws was calculated in moimix, why did the authors not actually calculate MOI? It would also be much clearer which samples should be excluded for the plot in Figure 1 with MOI estimates. The decision to use Fws rather than MOI needs to be clearly justified.  b. What was the cutoff for a sample to be considered a superinfection?  c. In Figure 1, the authors have filtered out samples with Fws < 0.85 but are then making a statistical comparison. How many samples were excluded? Could this exclusion affect the statistical analysis? Or was the statistical test run before excluding samples with lower Fws? Here again, MOI is more mathematically tractable than Fws as well as easier to interpret.  3. **Re: clustering.**  a. The ADMIXTURE data is very convincing and compelling. A PCA would also be beneficial to demonstrate further support for the geographic population structure.  b. The color scheme in Figure 2A is difficult to distinguish. The green and blue colors are insufficiently divergent in the context of this NJ tree. I would suggest the authors explore other, more divergent palettes in RColorBrewer or viridis.  4. **Re: IBD.** See my comment in the methods review. These cutoffs are not intuitive or consistent with the scheme outlined in the methods.  5. **Re: introgression.**  a. Figure 4C has the same problem as Figure 2A.  b. Are the proteins in Table 1 based on Pf orthologs (i.e. are they all putative)? I suspect that their biological significance is likely unclear based on my own experience with this type of analysis, but it wouldn’t hurt to be a little more explicit about the weight of the evidence here. | 1a. Human. The samples come from *P. knowlesi* infected patients.  1b. As suggested, we have added more detail on the depth and percentage of bases covered across both samples and chromosomes, giving ranges for both.  2a. The primary reason for using Fws instead of an MOI-based tool is that our focus was not on counting the number of clones but rather on determining whether the samples exhibit genetic complexity. This was necessary to exclude complex samples from further analysis. When multiple clones present in a sample are genetically similar, Fws will report it as having low complexity because it assesses allele frequency distributions. In such cases, the allele distribution appears homogeneous, as the strains are nearly identical. Since there is minimal genetic variation between the clones, their presence does not introduce bias into downstream analyses. In this context, the simple enumeration of clones is not beneficial.  However, to follow up on your comment, we did try binomix and found that it consistently overestimates the number of clones (based on Fws and NRAF plots), with binomix suggesting most samples have > 3 clones present. We have discussed this with close collaborators who have published on knowlesi previously, and they have experienced similar issues with other MOI tools. They hypothesise that it’s because these tools are designed for falciparum, which has considerably less complexity. Some tools allow thresholds to be tweaked, but this will take considerable time, and we think the value MOI would add does not outweigh the time investment.    2b. No cutoff was used to determine a superinfection. Initially, we used NRAF plots to distinguish superinfection vs co-transmission, suggesting that obligate recombination in the context of co-transmission would lead to complex diversity patterns in the plots. However, as pointed out by reviewers, this does not appear accurate, and we have amended the results as suggested.  2c. The analysis was run before filtering for Fws. We have clarified in the text that the analysis was performed prior to filtering and have fixed the plot, which appeared to be only of those samples that passed the Fws filtering threshold.    3a. We have added a PCA to the supplementary material.  3b. As the colour palette is already viridis and we want to keep the palette colour-blind friendly, we have instead increased the thickness of the lines to help with differentiation. We believe this provides better clarity.  4. We have amended the figure to align with what is described in your methods comment.  5a. Same response as 3b.  5b. They are annotations from the PKA1H1 reference genome but are all putative. We have added this information as suggested. |
| Reviewer #1 | Major | Conclusions | Please see ‘general comments.’ | NA |
| Reviewer #2 | Minor | Conclusions | No response. | NA |
| Reviewer #3 | Major | Conclusions | The conclusions overall are well supported, with clear limitations described. However, see my comment in the “Summary and General Comments” section about appropriately contextualizing the findings. | NA |
| Reviewer #1 | Minor | Editorial | Please see ‘general comments.’ | NA |
| Reviewer #2 | Minor | Editorial | No response. | NA |
| Reviewer #3 | Minor | Editorial | There are some missing words, typos, and missing italics throughout the manuscript. | We have proofed the paper again and made appropriate changes thank-you. |
| Reviewer #1 | Major | General Comments | This manuscript describes the addition of 52 whole genome sequences of Plasmodium knowlesi from Sabah state to a panel of previously sequenced genomes, and the in-depth population genomics analyses of the combined dataset. This well written account adds further insights to the current understanding of the population structure of Pk in Malaysia, and, the authors claim, reveals ‘genetic drivers of adaptation’ in this parasite. The manuscript describes complex population genetic analyses, which appear to give some fairly simple results; the previously described population structure of Pk in Malaysia holds up, and there are some mutations in drug resistance genes that were probably not selected by drug pressure. There is some evidence for introgression between the three populations, especially between the two populations previously characterised as representing distinct pools of parasites infecting Macaca nemestrina and Macaca fascicularis.  1.I have one comment/question regarding the authors assumption that an infection with two very different clones must be the result of super-infection rather than co-infection. What piqued my interest in this, was the observation that all their supposed ‘superinfections’ were composed of two distinct clones. Please forgive my naivety here, but I wonder if the authors might clarify how they are able to differentiate between a parasite isolate that contains two distinct and unrecombined clones (a mixture) and one that contains the products of a genetic cross between the two same clones.  To simplify this thought, if a monkey was infected with two distinct contemporaneously gametocyte producing strains of Pk, and was bitten by a mosquito, these would either undergo recombination in the mosquito resulting in 50% recombinant progeny and 50% ‘selfed’ parental strains, or they would not recombine, and the resulting sporozoites would be 50% of each parental strain. The latter scenario is unlikely, but could happen due to mating incompatibility or something.  However, when you sequence the resulting blood stage infection, you would measure the same thing – 50% alleles of parent A, and 50% alleles of parent B. How is it possible to differentiate between them in this case? What am I missing here? I think what I’m struggling (badly) to convey, is that just because there are two seemingly unrelated alleles at each locus in a 2-strain infection, it doesn’t follow that these must have come from a superinfection. A co-infection is just as likely, isn’t it? Please bear in mind I’m coming at this from experience of making genetic crosses between two different strains of parasite in the lab, and finding out if we’ve got a ‘cross’ or a ‘mixture’ is relatively taxing. In both cases, of course, it was not a superinfection, but a coinfection that produced the mixed allele infection. So, how confident are the authors that the 2-clone human infections are superinfections, and not just the result of a person being bitten by a mosquito that previously bit a monkey with a 2-clone Pk infection (that very well could have been the result of a superinfection, as I bet these are extremely common in monkeys). I raise this point because it is interesting to me as a geneticist and an epidemiologist, but also because it impacts on one of the main points of discussion for the paper. Please clarify.  2. Another, niggly (sorry) point. I think this paper expands our knowledge of what’s going on with Pk in Borneo, and commend the authors on carrying out some very clever work. But, to claim that the work is (or similar work could be) “invaluable in guiding future public health surveillance and control strategies” is reaching a bit, isn’t it? If such a claim is to be made that the results of this work can guide future control strategies, then this statement should be backed up with some real, solid examples of just how this will work. Which data/findings here will help control strategies? How will they do so? What differences in control strategies should be made after these results were generated compared to before? | 1. Thank-you for this insightful comment. The conclusion that the infections with distinct clones must come from superinfections was built on the assumption that there would be obligate recombination/mixing occurring in the mosquito, and so, two distinct clones could not be passed on in the context of co-infection. However, we now recognise that this is not entirely accurate and have amended the results and discussion to encompass other appropriate alternatives as suggested.  2. Thank you, we agree and have clarified our statement to avoid overestimating the direct public health translational aspects of this work. Indirectly we feel that continuing to grow our knowledge and adapting principles taken from Pf and Pv (eg. MalariaGen) to a zoonotic malaria context will hopefully assist in improved control strategies in the future, including the provision of initial evidence for the generation and possible validation of relevant case-use scenarios. For example, our initial design to combine genetic, vector and land use data in this analysis. Other examples could include using large collaborative WGS datasets to identify selection pressures such as drug resistance if sustained human-to-human adaptive transmission occurs, or the development of cost-effective molecular surveillance tools for ongoing genetic surveillance including markers of a spatially heterogenous disease burden. These kinds of strategies can be leveraged to consider novel approaches to integrating clinical, genetic and epidemiological/environmental surveillance for zoonotic malaria. |
| Reviewer #1 | Minor | General Comments | Further (extremely) minor points:  **Abstract**  Line 66 – please expand “IBD” here **Author Summary**  Line 84- I think it is very much the highest the highest burden in the country, and, indeed, the world.  Line 85. I think the word “thus” is not needed here, as it doesn’t really follow.  **Introduction**  Line 132. What gap? Suggest changing ‘this’ to ‘the’  Line 155: Perhaps mention the PCR target genes here to save a click for the reader?  Line 230 Any chance of a reference for Moran’s I?  Line 259: Same for Akaike Information Criterion. I appreciate these terms may be common and immediately understandable to modern population geneticists, but please be kind to the potentially more general readership of PLoS NTDs. In fact, the methodology in its entirety would benefit for some simple explanations of how the various methods actually work, and the concepts they’re based on, in layman’s terms. A hard task I know, but worth the effort!  Line 291: Could the dates ranges of collections be given here? Were samples collected contemporaneously for all populations? If not, is this a potential problem?  Line 301: What does ‘exclusion for minor allele frequency mean’? | Line 66. Expanded.  Line 84. Changed to world.  Line 85. ‘Thus’ removed.  Line 132. Reworded.  Line 155. Target added.  Line 230. Reference added.  Line 259. Reference added for AIC and several other analyses, with some brief one sentence descriptions also added. We focused on those that may be important for interpretation (rather than up-stream processing).  291. We have added the date ranges to the results. The samples were collected over a 6-year period. We don’t believe that this is a significant problem, as evidenced by the further substantiation of the major *P. knowlesi* clusters described from earlier periods across Malaysia including Sabah. However, we agree your suggestion to include the range here is beneficial, but it shouldn’t impact any major part of the findings from the analysis.  301. There is more detail on the MAF filtering in the methods with only a brief statement included in the results to avoid duplication. In brief, we filter out SNPs with low allele frequencies to remove potential errors, reduce noise, and reduce bias caused by rare variants. |
| Reviewer #1 | Major | Discussion | 1. Mainly, my concerns are with the assertion of superinfections, which I may be horribly wrong about.  2. Line 613 Why do you think these have ‘emerged’ (presumably you mean recently), and not been there for a long time?  3.The discussion is very long – I usually applaud this, but in this case it might be better to streamline it to get to the salient points and hammer home the main messages. It repeats a lot of what was already said in the results section. I’d like to see this section focus more on what the consequences of these findings are, practically, rather than rehashing the results. | 1. We have amended the discussion on superinfections, as stated above.  2. Line 613. This is an interesting point. We have no clear evidence to justify that these subpopulations within the established Mf and Mn clusters emerged recently, although cannot discount this given the known introgression events and increased parasite diversity associated with recent land use change and interaction across the macaque hosts/Pk clusters. Therefore we have simplified the sentence by removing “the emergence of”.  3. Taking all the helpful comments made by the reviewers into account, and being careful not to restate results and to be concise, we have rewritten much of the discussion. |
| Reviewer #2 | Major | General Comments | **Jacob Westaway and colleagues present a very comprehensive analysis of P. knowlesi whole genome sequencing data, combining 52 new genomes and 100 published genomes from different parts of Malaysia.** The manuscript is well written and relatively long, which is mostly justified due to the detailed analysis (though some suggestions to shorten and clarify the discussion are given). In some sections, there appears to be a bit of a risk of overinterpretation of the data, details below. In some other sections, some analysis (or their descriptions) would benefit from further clarification.  **Major comments**  1. Lines 320 to 327, and Figure 1B: The logic of the text and figure is difficult to follow. Unless I misread Figure 1B, the plots show the relative proportions of clones in multiclonal infections. The plot at the bottom left represents a multiclonal infection with an approximately 90%:10% split, while the plot at center right represents an approximately 50:50 split. These plots do not say anything about the relatedness between the clones. This is only captured in the Fws data, which is a very small part of Figure 1B. (I am not sure whether a correlation between Fws and NRAF is expected or typically expected – if there is, please apologize my ignorance).  2. For Figure 1A, the text states that it shows different relatedness levels, but the figure only ranges from 0.87 or so to 1.0. I am confused as to why the figure does not capture the full range of Fws values observed.  3. Lines 387-88: The following sentence is not correct: “At IBD thresholds under ~30%, three relatively distinct, large infection networks were observed” At 11.8%, there are two networks, with the remaining genomes not being connected. It is not correct to define them as a network.  4. Lines 290-92: Am I supposed to see that in Figure 3? I am not sure whether the lines are invisible, or whether it is not displayed. (Edit: The figure legend answers this question (not shown as <11.8%), but still the main text could be clarified).  5. Also, it seems that at such a low threshold (3%), genomes are connected almost always. Is this truly a result, or simply an artifact? Could you run a statistical test on that? It seems unusual to comment on anything being related at the 3% threshold. | 1. The word related was used as an antonym for distinct (clones). We have replaced it with more precise terminology. To be clear, NRAF plots with distinct bands at multiple allele frequencies suggest the presence of distinct (no mixing) clones, whereas more complex patterns represent genetic mixing.  2. This was an error and has been amended.  3. Statement removed and paragraphed restructured to accommodate.  4. No, it is not presented in Figure.  5. We have removed this result. |
| Reviewer #2 | Major | Methods/Results | Lines 498-509 (and lines 695-690): I have a number of questions about this analysis. First, the hypothesis that introgressions are directly linked to vector habitats is far-fetched. Introgressions likely happened many transmission cycles before the sample was collected for sequencing, it is unknown whether at this point the parasite was transmitted in a similar habitat.  Second, parasites collected closely together are expected to be closely related and likely carry the same introgressions, but are also more likely to share habitat characteristics. It appears to me that this confounder was not accounted for. Chi square tests require independence between observations. This was likely not the case here, resulting in invalid results. In my opinion, this analysis is error prone and adds little to the manuscript, and thus could be deleted. | To your first point, we agree that we cannot precisely determine the timepoint at which introgression events have occurred and have now commented on the limitations around this further in the discussion. However, we believe that underlying localised transmission intensity influences the risk of introgression events occurring and is consistent with an increasing evidence base demonstrating how recent environmental forest fragmentation is driving vector abundance and host biting preferences across macaque species with previously separate Pk subpopulations. Therefore, we feel that this remains an interesting and valid hypothesis worth testing in this analysis despite the inherent limitations. The vector habitat suitability model we applied is based on inferences around composite measures of various environmental land classification features (therefore generalisable across Sabah) at a spatial scale consistent with factors such as the presence of vector breeding sites, the geographical range of macaque troops and occupational human movement data for those working in nearby areas.  To your second point, we tested and reported the environmental and vector factors for spatial autocorrelation using the well validated Moran’s I (the degree to which objects/values that are close together in space are more similar or dissimilarly than those further apart) prior to the analysis and found that there was no correlation. The habitat characteristics are based on a 5km x 5km grid around the collection site, and these landscapes differ given the 10m resolution used to calculate a number of substantially different environmental land classification metrics over these distances.  However, you are correct in that the confounder of spatial distance being associated with genetic relatedness (and thus more likely to share the same introgressions) was not accounted for. Thus, to determine if this was indeed a confounder of the analysis, we calculated pairwise distances (Haversine method) between GIS coordinates and performed a Spearman’s Rank Correlation analysis between this value and pairwise IBD. There was no significant association between spatial distance and genetic relatedness (S = 49670951, p-value = 0.8194) |
| Reviewer #2 | Major | Methods/Results | Lines 515-543 and Figure 5: This section seems to be overlapping Figure 2B and C and associated texts. To improve clarity of the manuscript, I recommend combining these sections and discuss the distribution and structure of Mf and Mn in Sabah and Sarawak only once.  Line 704: “…suggests introgression events could be arising independently” In that case, would they have slightly different break points? If so, could you check whether this hypothesis is true? | Although there is overlap with Figure 2B/C (same type of analysis), Figure 5 and its associated analysis, along with the following fixation analysis (Figure 6), was performed on the genomic clusters (Mf and Mn) individually. Thus, to improve clarity we have moved these sections to follow on from the initial IBD, as you suggest, but have kept them separate (with sub-headings) to make it clear that the two IBD analyses are different, and we have expanded the description of this difference in text.  Line 704: The introgression analysis was done using genetic distances within sliding windows (10kb) and so we can’t identify the specific breakpoints. However, we plotted the windows across sections of chromosomes for isolates from different states and observed that although the introgressed regions overlap (groups of windows), the window at which regions start are different. Suggesting the breakpoints are in different positions and that the same events are arising independently. We have added an example plot to the supplementary material to support this and made reference to it in the text. |
| Reviewer #2 | Minor | Line-specific comments | 1. Lines 60 and 85: Given Malaysia has among the highest Pk transmission in the world, would it be more adequate to say that in Sabah it is among the highest in the world?  2. Line 102: It seems a word is missing here. Maybe “In contrast to the control of other species…”  3. Line 105: “Plasmodium species” used above (not “Spp”), recommended to be consistent.  4. Line 135: Please add that out of the 108 genomes, 100 were included in the analysis.  5. Line 309: The title “High prevalence of polyclonal P. knowlesi infections in Malaysian Borneo, but infrequent superinfection” seems not to represent the data. First, I would question whether 10-17% is high. Second, the frequency of polyclonal infections is virtually identical in peninsular Malaysia and in Borneo, thus way stress out Malaysian Borneo? Following this, lines 316-318 are confusing: “The highest proportion of polyclonal infections was observed in Sarawak (17.6%, n=13/74), followed by Sabah (10.6%, n=10/94), and Peninsular Malaysia (12.1%, n=4/33).” 12.1% is more than 10.6%, thus Sabbah does not follow Sarawak.  6. Lines 352-53: There is no possible way to split 16 genomes to come to 86.6% and 13.4%. Please check these numbers.  7. I recommend making figure 2B larger. It is hard to see the dots on the map, and the legend next to it is also hard to read. | 1. Corrected from country to world.  2. Revised as suggested.  3. Changed to species for consistency.  4. Added that 100 were included in the analysis.  5. We have removed the statement of high prevalence and corrected the order of state specific values.  6. Left a digit off – corrected to 164.  7. Have made Figure 2B larger. |
| Reviewer #3 | Major | General Comments | **Westaway and colleagues present a very interesting and convincing analysis of the genomics of an emerging and neglected malaria pathogen. This paper merits publication and is a valuable contribution to the literature.**  Strengths: Appropriate analyses are used, compelling and clear results are presented, and the data is well situated with regard to Pk biology. The writing is clear, and the figures are well-organized.  Weaknesses: Overall this is a strong paper. The weaknesses are largely related to unexplained methodological choices, as outlined in specific sections.  **Major point:**  1. The authors consistently refer to evidence from other Plasmodium species as they attempt to interpret biological significance in P. knowlesi. As authors consistently refer to evidence from other *Plasmodium* species as they attempt to interpret biological significance in *P. knowlesi*. As a researcher used to working on non-model organisms, I strongly empathize. However, this evidence is not put into sufficient context. *Plasmodium knowlesi* is a distinct organism, which is not particularly closely related to any of the other *Plasmodia* from which evidence is derived. It should be made much more explicit throughout the results and discussion that results from *falciparum* or *berghei* in particular are not easily translated into biological significance in *knowlesi*. It is good and appropriate to mention this evidence from other species as the authors make their interpretation, but they should consistently acknowledge that the biological significance in *Pk* is unknown and cannot be assumed to be identical to that in culturable species with different natural hosts.  2. In addition, **re: drug resistance** in particular, the putatively resistant alleles in *Pv* need to be put into appropriate context, as being either purely association-based or based on orthologs in *Pf*. Therefore, since we cannot even assume that they play a biologically significant role in resistance in *Pv*, the connection to biological significance in *Pk* is even more tenuous. The authors are appropriately cautious in interpretation, but should soften their language even further, since there is no compelling empirical evidence for a role in *Pk* drug resistance. | 1. We have added that genes are putative, where appropriate, and made several statements in the results and discussion suggesting caution be taken when interpreting the results as the biology may not translate from other *Plasmodium* species to *P. knowlesi*.  2. As suggested, we have softened the language in the results and around the interpretation in the discussion (adding a few caveats). We have also included some more context to the *P. vivax* “resistant” markers. |
| Reviewer #3 | Major | Methods/Data Availability | **Minor point:** I attempted to locate the uploaded sequence data on SRA, but was unable to find any record of it. I assume there was a typo or other minor mistake, but this needs to be rectified before publication. | The automatic data release has been set up through SRA to occur when the manuscript is published or in 2025, whatever comes first. |
| Reviewer #2 | Major | Methods | **Lines 387-388:** The statement “At IBD thresholds under ~30%, three relatively distinct, large infection networks were observed” is incorrect. At 11.8%, there are two networks, with remaining genomes not being connected. It is not correct to define them as networks. | Statement removed and paragraph restructured to accommodate. |
| Reviewer #2 | Major | Results | **Lines 498-509** (and **Lines 695-690**): The hypothesis that introgressions are linked to vector habitats is far-fetched. Introgressions likely occurred many transmission cycles ago. Chi-square tests require independence between observations, which is not the case here. This analysis should be reconsidered or removed. | Please see response to the reviewer’s previous comment on this. |
| Reviewer #2 | Major | Discussion | **Line 704**: “suggests introgression events could be arising independently.” If this is true, the breakpoints should differ. Check this hypothesis. | The analysis was done using the genetic distance across 10K bp windows, and so it cannot be used to identify specific breakpoints. However, we have added a plot to the supplementary material that compares isolates from different states and the distribution of windows across a heavily introgressed region, showing a different distribution of windows, supporting this statement. |
| Reviewer #3 | Minor | Figures | The color scheme in **Figure 2A** needs improvement for clarity. The blue and green are hard to distinguish. Consider using **RColorBrewer** or **viridis** for a more divergent palette. | Please see response to the reviewer’s previous comment on this. |
| Reviewer #2 | Minor | Results/Figures | **Lines 352-353**: There is no way to split 16 genomes into 86.6% and 13.4%. Please recheck these numbers. | Corrected to 16**4**. |
| Reviewer #2 | Minor | Results | **Line 659:** The use of “unfortunate” to describe individuals inoculated multiple times seems unscientific. Please rephrase. | Unfortunate removed. |
| Reviewer #2 | Minor | Results | **Line 724:** The term “genetic transfer” should be changed to “introgression” for clarity. | Corrected. |
| Reviewer #2 | Minor | Discussion | **Lines 750-752:** The sentence regarding the N272S mutation within the *dhfr* gene should be clarified. Is the mutation highly prevalent or only found in lab-adapted lines? These seem mutually exclusive. | Rephrased for clarity. |
| Reviewer #2 | Minor | Discussion | **Lines 755-757:** This sentence suggests SP “treats” infections, but SP kills the parasite. Rephrase for clarity. | Have rephrased for clarity. |
| Reviewer #3 | Minor | General Comments | There are some small issues with missing words, typos, and missing italics throughout the manuscript. For example, in **Line 84**, *P. knowlesi* is not italicized. Similarly, in **Lines 364-365**, a word appears to be missing: “evidence of shared ancestry.” Double-check the manuscript for other similar errors. | Corrections made for specific examples, and manuscript has been checked with amendments made where needed. |
