# Supplementary material for: Genomic epidemiology of Plasmodium knowlesi reveals putative genetic drivers of adaptation in Malaysia
Source: PLoS Negl Trop Dis. 2025 Mar 12;19(3):e0012885. doi: 10.1371/journal.pntd.0012885 (PMC11932472; doi:10.1371/journal.pntd.0012885)
Supplement: Attachment — Submitted filename: Reviewer_comments.docx [file pntd.0012885.s004.docx]

| **Reviewer** | **Comment Type** | **Manuscript Section** | **Comment** | **Author** |
| --- | --- | --- | --- | --- |
| Reviewer #1 | Minor | Methods | No response. | NA |
| Reviewer #2 | Minor | Methods | No response. | NA |
| Reviewer #3 | Minor | Methods | No response. | NA |
| Reviewer #1 | Minor | Results | No response. | NA |
| Reviewer #2 | Major | Results | **The revised manuscript is greatly improved. A few points remain that should be addressed to include clarity:**  1. Please add line numbers to future versions of the manuscript.  2. **Paragraph 1 vs. Paragraph 2 of Results:** The first paragraph of the results explains the composition of the 152 samples included in the analysis. The second paragraph then presents data based on 201 isolates. It is difficult to understand why additional isolates were included in this analysis.  3. **Figure legend 1:** While a Manhattan Plot is not strictly defined, and thus technically it might not be wrong, the plot in this figure is rather different from a typical Manhattan Plot (e.g., used in a GWAS). I recommend not to use the term here.  4. **Introgression windows:** When reporting introgression windows, it is unclear whether the authors refer to the number of introgressions (i.e., biological events), or to the number of 10kb windows. For example, the authors state: “For the Mf cluster, chromosomes 8 (n = 35) and 11 (n = 21) had the greatest number of candidate windows, with several windows on chromosome 8 overlapping the large peak observed in the Fst analysis (Figure 5).” Does this mean there were 35 windows on chromosome 8 that were not adjacent, pointing to 35 independent introgression events? Or, is there a single window of 350kb, thus spanning 35 of the 10kb windows defined by the authors? If the latter, this is simply a result of the window size selected by the authors and does not have any biological meaning; rather the number of biological introgressions should be reported. Similar unclarity appears in other sentences.  5. **Comparison of sites:** “For Betong samples 85% (n =12/14) had high levels of introgression, compared to 50% of Papar samples (n=1/2), with all but one Betong sample from the Mf cluster.” Is it really worth adding a comparison if one site has only two samples? Naturally, no conclusions can be drawn from this statement.  6. **Satellite images:** From what year were satellite images to study habitat suitability? Given samples were collected from 2011-2016, and the fast-presumably deforestation in this part of the world, it seems important to use images from the time of sample collection. Please add the date of the satellite images, and maybe a comment on whether deforestation might have changed since samples were collected.  7. **Page 27:** What does “including 20 at high levels” mean?  8. **Page 27:** There seems to be a word missing in “suggesting that introgression events may be a contributing factor to regions of IBD.” Perhaps “High IBD”? | 1. Noted.  2. Apologies for the confusion. All 201 samples underwent Fws analysis, with additional filtering applied for remaining analyses. We have updated the first paragraph of the results to state this clearly: “F_WS_ analysis was performed on 201 samples, with additional filtering based on clonality, missingness, and minor allele frequency, used to generate a subset for other analyses. 52 of the newly sequenced genomes remained after the additional filtering, and another 100 genomes from the publicly available data.”  3. Have changed to dot plots.  4. We thank the reviewer for their comment and agree that the wording in the Results and Discussion sections could be improved for clarity. Our aim was to detect both recent and ancient introgression, which is why we used a small window size. This approach allows us to capture even the small fragments of introgressed segments that remain after many rounds of recombination. While recent introgression can span thousands to tens of millions of base pairs, ancient introgression is often fragmented due to the effects of recombination over time. Factors such as recombination rate, the number of generations since the event, and potential selection on introgressed segments influence segment length.  As noted, our analysis reports 10 kb windows rather than “biological windows.” Defining a single biological introgression event is challenging because grouping adjacent windows can introduce ambiguity, as outlined above. For example, a cluster of windows in a genomic region showing multiple start and stop positions across samples (see the last page for an example) could reflect either a highly recombined ancient introgression event or several smaller, more recent introgression events. Given this complexity, we chose to report the technical windows without making assumptions about the underlying biology.  To address the reviewer’s concern, we have revised the Results and Discussion sections to ensure consistent terminology and explicitly state that we are referring to 10 kb windows, not biological windows. Added on line 505: “Given the complexity of distinguishing single biological introgression events, due to potential ambiguity when grouping adjacent windows with varying start and stop positions across samples (Supplementary Figure 8), we define the 10kb windows identified in our analysis as introgressed windows without making assumptions about the underlying biological events”. We have added the figure at the end of this document to the supplementary outputs and referred to in the main text of the manuscript. This should improve clarity and avoid confusion.  5. We agree that a comparison is unnecessary, but we think that it’s worth stating the proportion and number of samples that appear to be highly introgressed, especially given there are only two samples from Papar. I.e. given the low number of samples from Papar, readers should not read into it having the second highest average introgressed windows. We have restructured the sentence to reflect this: “85% (n =12/14) of samples from Betong and 50% (n=1/2) from Papar had high levels of introgression, with all but one Betong sample from the *Mf* cluster.”  6. We have stated in the methods that the composite landscape metrics tool we used is based on the ESRI 2020 release and have now added the sampling dates for the mosquito layers. The mosquito vector layer is a predicted distribution based on sampling between 2011-2016, so, with quite significant overlap with the sampling for the genomics. Although not perfect, it is the best marker of species distribution available. We also have a comment in the discussion, but have expanded it to align better with your comment: “However, this integrated genomic and spatial analysis is limited by the small subset of isolates and landscape metrics used (82), as well as the lack of temporal alignment between *P. knowlesi* isolate collection and environmental data, especially when one considers ongoing deforestation, reforestation, as well as land use change in Sabah.”  7. This was in reference to the previous sentence, where we define a high level of introgression as >5 windows. However, we have amended the sentence for clarity: “…including 20 with >5 windows.”  8. No word is missing, but we can see how this could be confusing/misleading. We have added a few words for clarity: “…suggesting that introgression events may be a contributing factor to shared regions of IBD between samples.” |
| Reviewer #3 | Minor | Results | No response. | NA |
| Reviewer #1 | Minor | Conclusions | No response. | NA |
| Reviewer #2 | Minor | Conclusions | No response. | NA |
| Reviewer #3 | Minor | Conclusions | No response. | NA |
| Reviewer #1 | Minor | Editorial | No response. | NA |
| Reviewer #2 | Minor | Editorial | No response. | NA |
| Reviewer #3 | Minor | Editorial | No response. | NA |
| Reviewer #1 | Minor | General Comments | No response. | NA |
| Reviewer #2 | Minor | General Comments | No response. | NA |
| Reviewer #3 | Minor | General Comments | **I appreciate the authors’ thoughtful responses to my and the other reviewers’ comments. The figures are also much improved.** **Two very minor issues:**  1. The way the authors refer to identity by descent and identity by state needs to be standardized. Throughout the manuscript, it is variously written in all caps, all lowercase, or all lowercase with hyphens (which I believe is the most correct, but the decision is yours). There are also multiple instances where descent is misspelled as either “descent” or “decent”.  2. “join-genotyping” should presumably be “joint-genotyping.”  **One comment for future consideration:** Re: clonality, it is well known that many of the COI/MOI estimators spuriously inflate the number of clones present in a given sample, but this issue is not insurmountable. I am fine with your paper proceeding as-is with Fws since it’s a minor finding, and might be useful for your future work: I exclusively work on non-falciparum species (though not Pk), so I don’t buy the idea that Pk is uniquely unsuited to these methods. Filtering by WSAF, missingness, and coverage (specifically with samples that are high outliers) can help alleviate the overinflation issue. I have also generated more reasonable estimates with coiaf than with The Real McCoil, but both are robust and I have used both for non-falciparum species. | 1. We have standardised to the suggested lower case with hyphens.  2. Corrected.  We also appreciate the final comment as this will be important for future work, where we have plans to compare the number of clones between different infection types. We will explore filtering parameters to try and improve clonal estimates. |

**Supplementary Figure 8.** Tile plot of introgression windows (10kb) across chromosome 8 for all *Mf* samples, where introgressed windows are designated *Mn* (more genetically similar to *Mn* than *Mf*), demonstrating the level of fragmentation across highly introgressed regions and highlighting the difficulty in assigning biological window
